# Correction: The implementation and impact of non-invasive prenatal testing (NIPT) for Down’s syndrome into antenatal screening programmes: A systematic review and meta-analysis

**DOI:** 10.1371/journal.pone.0318985

**Published:** 2025-02-04

**Authors:** Elinor Sebire, Chithramali Hasanthika Rodrigo, Sohinee Bhattacharya, Mairead Black, Rachael Wood, Rute Vieira

There are errors in the Author Contributions. The correct contributions are:

**Conceptualization:** Elinor Sebire, Sohinee Bhattacharya, Rachael Wood, Rute Vieira

**Data curation:** Elinor Sebire, Chithramali Hasanthika Rodrigo, Rute Vieira

**Formal analysis:** Elinor Sebire, Rute Vieira

**Funding acquisition:** Sohinee Bhattacharya, Rachael Wood, Rute Vieira

**Investigation:** Rute Vieira, Elinor Sebire

**Methodology:** Elinor Sebire, Chithramali Hasanthika Rodrigo, Sohinee Bhattacharya, Rute Vieira

**Project administration:** Elinor Sebire, Rachael Wood, Rute Vieira

**Supervision:** Sohinee Bhattacharya, Mairead Black, Rachael Wood, Rute Vieira

**Visualization:** Elinor Sebire

**Writing–review & editing:** Elinor Sebire, Chithramali Hasanthika Rodrigo, Sohinee Bhattacharya, Mairead Black, Rachael Wood, Rute Vieira

**Writing–original draft:** Elinor Sebire

The ORCID iDs are missing for the second, third, fourth, and fifth author. Please see the authors’ respective ORCID iDs here:

- Author Mairead Black ORCID iD is: 0000-0002-6841-8601
  (https://orcid.org/0000-0002-6841-8601)
- Author Chithramali Rodrigo ORCID iD is: 0000-0002-0524-494
  (https://orcid.org/0000-0002-0524-494)
- Author Rute Vieira ORCID iD is: 0000-0002-3599-7299
  (https://orcid.org/0000-0002-3599-7299)
- Author Rachael Wood: 0000-0003-4453-623X
  (https://orcid.org/0000-0003-4453-623X)
- Author Sohinee Bhattacharya ORCID iD is: 0000-0002-2358-5860
  (https://orcid.org/0000-0002-2358-5860)
