# Supplementary figures and images for: The implementation and impact of non-invasive prenatal testing (NIPT) for Down’s syndrome into antenatal screening programmes: A systematic review and meta-analysis
Source: PLoS One. 2024 May 16;19(5):e0298643. doi: 10.1371/journal.pone.0298643 (PMC11098470; doi:10.1371/journal.pone.0298643)

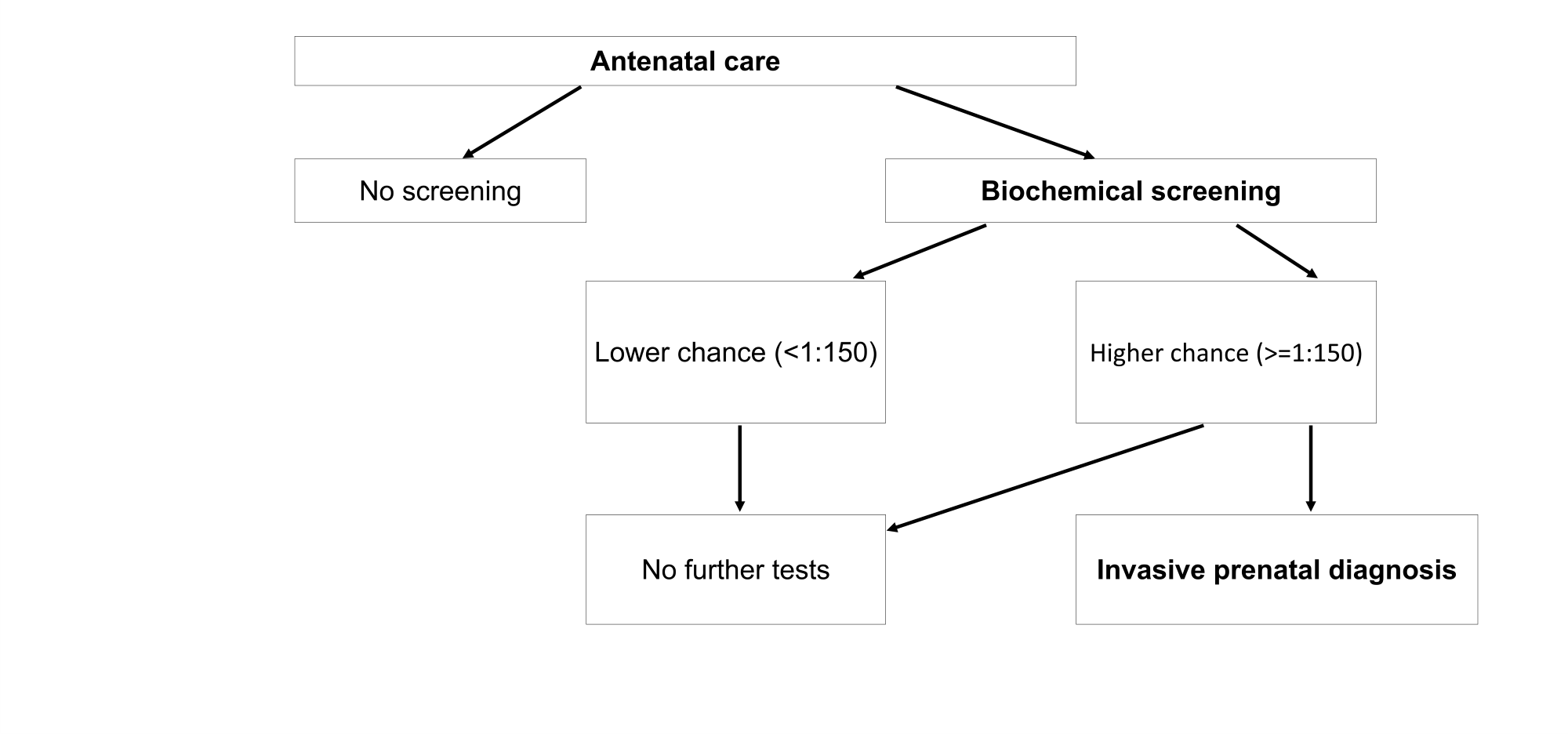

Supplement: S1 Fig — Also common to other countries with a routine screening programme. Timing of the screening tests, blood markers measured and threshold for ‘higher chance’ pregnancy may vary between health systems. Invasive prenatal diagnosis = amniocentesis or chorionic villus sampling. * The higher chance threshold is calculated as 1 in X pregnancies, with thresholds being set for progression to invasive prenatal diagnosis. NT = nuchal translucency. (TIF) [file pone.0298643.s001.tif]
