# Supplementary material for: The implementation and impact of non-invasive prenatal testing (NIPT) for Down’s syndrome into antenatal screening programmes: A systematic review and meta-analysis
Source: PLoS One. 2024 May 16;19(5):e0298643. doi: 10.1371/journal.pone.0298643 (PMC11098470; doi:10.1371/journal.pone.0298643)
Supplement: S1 Table — Table 1 of all studies included in this systematic review, along with Down’s and Black quality assessment score. 2. Search strategy, keywords and mesh terms used in database search. (DOCX) [file pone.0298643.s002.docx]

# S1 Table - Appendix 1

1. **Characteristics of included studies (parts A and B)**

| **Study ID** | **Study design** | **Aims** | **Time period of study** | **Part A - Country / state / province**  **Part B – population** | **Primary outcome measures** | **Funding source** | **Declarations of interest?** | **Down's and Black quality assessment score** | **Systematic review section** |
| --- | --- | --- | --- | --- | --- | --- | --- | --- | --- |
| Akaishi et al., 2015  (1) | Retrospective cohort study | Number of invasive confirmatory tests following NIPT, changes in the number of women seeking prenatal screening. | 2011-2014 | Hokkaido University Hospital, Japan | Number of women undergoing screening, diagnostic tests. | Not reported | Not reported | 14 | B |
| Bowden et al., 2022 (2) | Retrospective cohort study (observational) | To evaluate the implementation of MIPT on pregnant women’s choices in national NHS antenatal screening programme - uptake of NIPT, performance, and invasive procedures performed. | 2018 - 2020 | Wales, UK | Uptake of NIPT, performance of NIPT, and invasive procedures performed. | Public Health Wales | No conflicts of interest | 20 | A + B |
| Bjerregaard et al., 2017(3) | Retrospective cohort study | Investigate the effect of implementing NIPT among high-risk pregnancies in a region with first trimester screening for T21. | 2011 – 2015 | North Denmark region | Invasive testing rate among high-risk pregnancies pre and post NIPT | No funding received | Not reported | 22 | B |
| Chen et al., 2022(4) | Retrospective cohort study | determine the PV of expanded NIPT for chromosomal abnormalities in the second semester. | 2017 - 2021 | Shanghai, China | screening PPV and NPV for various age sub intervals and conventional indications | Not reported | No conflicts of interest | 19 | B |
| Choe et al., 2020 (5) | Clinical practise guidelines for NIPT screening | Guidelines for aneuploidy screening and diagnostic testing in Korea. Article outlining the clinical practise guidelines defined by the Korean Society of Maternal-Fetal Medicine. | \ | Korea | \ | \ | \ | \ | A |
| Dap et al., 2022(6) | Retrospective single centre study | Examine the use of NIPT in highest risk group (1:50) after first trimester serum screening | January 2015 - December 2020 | France - single centre (Nancy university hospital) | patient characteristics, test results and pregnancy outcomes | No funding | No conflicts of interest | 20 | B |
| Dougan et al., 2021(7) | Retrospective cohort study | Describe the population-based performance of Ontario's prenatal screening programme, with publicly funded cfDNA for some indications. The effect of introducing cfDNA on the prenatal screening and diagnostic choices made by women. Using available data on BORN registry. | Sep 2016 - Mar 2019 | Ontario, Canada | Sensitivity (DR), specificity and screen positive rate for T21 and T18 ; Uptake of prenatal screening for T21 ; Number of positive multiple marker screened pregnancies that went on to have cfDNA or invasive prenatal testing; The number of positive cfDNA pregnancies that went onto have invasive prenatal testing; Rate of screened pregnancies (proportion of all screened pregnancies) that underwent invasive testing from 2012 - 2013 cohort. | Supported in part by BORN Ontario, which is funded by the Ontario Ministry of Health. Heather Howley is supported by the CHEO Foundation. Mark Walker is a recipient of a Canadian Institutes of Health Research Foundation Grant (FDN No. 148438) | Nan Okun reports funding from Roche Diagnostic, outside the submitted work. No other competing interests were declared | 23 | A + B |
| Duvillier et al., 2021(8) | Retrospective cohort study | To study the effect of implementing NIPT in France on the gestational age at termination | 2012 - 2017 | Poissy Saint-Germain Hospital, France | Median GA at termination; median GA for termination of trisomy 21; frequency of invasive procedures; median GA at the time of invasive procedures. | Not reported | No conflicts of interest | 22 | A |
| Flock et al., 2017 (9) | Prospective multicentre study | Evaluate the performance of rMPS (random massively parallel sequencing) for trisomy 21, 13 and 18 in routine clinical practise. And rate of invasive diagnostics. | 2013 - 2015 | German centres providing prenatal care - trialled by the University of Bonn. | demographic and clinical data of participants, test quality for T21, 13, 18. | LifeCodexx | No conflicts of interest | 22 | B |
| Gadsboll et al 2020*(10) | Survey of clinical providers in Europe, Australia and USA | Describing the current use of NIPT, access to NIPT, chromosomal coverage, financial coverage and proportion of women using NIPT. |  | Poland, Romania, Iceland, Lithuania, France, Italy, Finland, Norway, Slovenia, Wales | Survey to assess four areas of NIPT implementation in each country: NIPTs chromosomal coverage, NIPT's financial coverage, proportion of women receiving NIPT. Where no national data was available for proportion of women receiving NIPT, the survey asks for best clinical estimate. | Not reported | One author receives research support from Illumina and Natera. The funding went directly to the department. All other authors declared no conflict of interest. | 12 | A |
| Garshasbi et al., 2020(11) | Prospective nationwide multicentre observational study | To report the clinical experience and performance of NIPT in a mixed risk population in Iran. | 2015-2016 | 150 medical centres in Iran | sensitivity, specificity of the NIPT test. | Not reported | No conflicts of interest | 24 | B |
| Health and social services Yukon, Canada - Congenital Anomalies Support Yukon (12) | Patient decision aid for prenatal screening | describing the process and options available for prenatal screening in Yukon, Canada | Produced in 2019 | Yukon, Canada | None - patient information | Not reported | No conflicts of interest | \ | A |
| Hill et al., 2017 (13) | Retrospective audit of pregnancy outcomes | Analyse pregnancy outcomes for a trial period of NIPT implementation as a second line screening test. | March 2015 - October 2016 | North East Thames Region, UK | termination rates (as a proportion of all pregnancies that had high risk NIPT) | NIHR | One author is used by charity Antenatal results and choices. | 11 | B |
|  |  | Analyse the pregnancy outcomes for women who chose to have NIPT or went straight to invasive testing without prior NIPT. | April 2014- January 2017 | National University Hospital, Singapore | Termination rates (as a proportion of all pregnancies that had high risk NIPT), termination rates for women going directly to invasive testing without NIPT. |  |  |  |  |
| Hsiao et al., 2022(14) | Retrospective cohort study | To evaluate the impact of prenatal screening tests on invasive procedures and to survey the effects of prenatal screening tests on trisomy 21,13 and 18 detection rates. s and the proportion of advanced maternal age pregnancies. | 2006 - 2019 | Taiwan | Number of prenatal diagnosis procedures performed; trisomy 21,18 and 13 detection rates. | No funding received | No conflicts of interest | 22 | A + B |
| Kou et al., 2016(15) | Retrospective cohort study | To determine whether the introduction of NIPT influenced the IPD indications and prenatal detection of Down's syndrome | 2010 - 2013 | Queen Elizabeth Hospital, Hong Kong | Invasive prenatal diagnostic rate, false positive rate, prenatal detection rate of Down's syndrome | Not reported | No conflicts of interest | 17 | A + B |
| Kudryavtseva et al., 2022(16) | Retrospective cohort study | Evaluate the effectiveness of NIPT for high chance pregnancies for Down’s syndrome. | January 2013 – December 2018 | Ural State Medical University and ‘Genomed’ Medical genetic centre (Russia) | NIPT and invasive prenatal diagnosis results in higher chance women, post-natal examination of baby and follow up. | No funding received | No conflicts of interest | 19 | B |
| Lindquist et al., 2019(17) | Retrospective cohort study | performing record-linkage study to ascertain the real world use and performance of various screening pathways and report the numbers of major chromosomal conditions detected after a low risk CFTS or cfDNA result. | 2015 | Victoria, Australia | Post-natal cytogenetic results from pregnancies and infants up to 12 months obtained to ascertain false-negative screening results and abnormal results. | National Health and Medical Research Council Early Career Fellowship and Senior Research Fellowship. | Two authors report commercial relationship with Roche Diagnostics and Natera, and personal fees from Phillips Ultrasound. | 23 | A+B |
| Liu et al., 2021(18) | Retrospective cohort study | Analyse the clinical application of NIPT based on second trimester ultrasonographic soft markers in low-risk women. | 2015 - 2019 | West China second university hospital | Performance of NIPT in detecting foetal aneuploidies, clinical follow up of high and low risk screening pregnancy outcomes. | Not reported | Not reported | 20 | B |
| Lund et al., 2020(19) | Retrospective cohort study | Evaluate the early clinical use of NIPT in Danish public and private healthcare settings before NIPT guidelines are introduced in 2017 | 2013 - 2017 | Denmark | Collect and describe the early clinical use of NIPT in Denmark in both public and private settings before national guidelines were introduced in 2017 | Not reported | No conflicts of interest | 22 | A + B |
| Manegold-Brauer et al., 2014(20) | Retrospective cohort study | Describe the current impact of NIPT on prenatal care. | 2011 - 2013 | University Hospital Basel (Switzerland) | Change in invasive test use after FCT vs NIPT. | No funding received | No conflicts of interest | 17 | B |
| Manotaya et al., 2016(21) | Prospective large multicentre cohort study | To report the clinical experience and performance of NIPT as screening for trisomy 21,18,13 in mixed risk population in Thailand. | 2012 - 2014 | 121 medical centres in Thailand | The performance of NIPT, and incidence of T21,18 and 13. comparing NIPT detection of trisomies in high risk and low risk groups. | Not reported | Not reported | 19 | B |
| Martinez-Payo et al. 2018(22) | Retrospective cohort study | analyse the introduction of NIPT into contingent screening for T21 | 2012-2013 vs 2016 | Puerta de Hierro University Hospital, Madrid, Spain | Comparison of before and after NIPT introduced. | Not reported | Not reported | 19 | B |
| McLennan et al., 2016 (23) | Cohort study | To describe the tests performed in 2 private practises where NIPT has been implemented as a first line and contingent screening test | 2013-2014 | Three private practises in Australia | Number of women positive after first and contingent screening. Detection rate of trisomies in the cohort. | Not reported | Not reported | 20 | B |
| Norwegian Health Directorate - national professional guidelines *(24) | national guidelines for prenatal screening offered | \ | \ | Norway | \ | \ | \ | \ | A |
| Olenev et al., 2021  (25) | Prospective cohort study | assess the effectiveness of including NIPT in the structure of prenatal diagnostics in Moscow. | April - September 2020 (5 months) | Russia | NIPT results in different risk groups, performance of NIPT, age of women and positive NIPT results comparison. | Moscow Health Department | No conflicts of interest | 17 | A |
| Public Health England - 'Screening for Down's syndrome, Edward's syndrome and Patau's syndrome: non-invasive prenatal testing (NIPT). Sep 2021  (26) | Screening guidelines | Operational guidance for people offering NIPT for t21, T18 and T13 | \ | England | Eligibility for NIPT, laboratory requirements for NIPT, blood sampling and transport, reporting NIPT results. | \ | \ | \ | A |
| Sainz et al 2020 (27) | Prospective cohort study | Assess the performance, patient uptake, and cost of contingent cfDNA testing compared to established FCT screening. | 2016 - 2018 | South Spain - two centres | uptake of NIPT and choices made. detection rate, false-positive rate, patients uptake and associated costs evaluated. | No funding | No conflicts of interest | 24 | B |
| Samura et al., 2017(28) | Retrospective cohort study | Report the 3-year experience of a nationwide demonstration study of NIPT and review the current status of NIPT in Japan. | 2013 - 2016 | Japan | Clinical data, test results and pregnancy outcomes recorded. | Grant from national centre for child health and development | No conflicts of interest | 18 | B |
| Sasaki et al., 2021(29) | descriptive cross-sectional study | analyse the clinical performance of NPIT in a single laboratory. | 2015 - 2019 |  | sensitivity, specificity, and positive rate of the NIPT lab test. | Japanese ministry of health, labour and welfare. | No conflicts of interest | 16 | B |
| Scottish Government - Chief Medical officer Directorate Aug 2020(30) | Chief Medical officer directorate | Letter from the chief medical officer setting out the changes to the Scottish pregnancy screening programmes. | \ | Scotland | \ | \ | \ | \ | A |
| SFOG guidelines 2016 Guidelines; NIPT for trisomy 21,13,18(31) | Screening guidelines | Described NIPT testing for trisomy 13, 18 and 21 with clinical recommendations. | \ | Sweden | Recommendations and evidence for the clinical implementation of NIPT for trisomy screening. | \ | \ | \ | A |
| Shah et al., 2014 (32) | Prospective cohort study | Analyse the options chosen by women after cfDNA introduced into the screening programme. | 2011 - 2012 | San Diego Thornton Hospital, University of California | Impact on testing chosen, by trimester, health insurance coverage and ultrasound abnormalities. | Data by the foetal care centre., university of California. | No conflicts of interest | 22 | A+B |
| Swiss public health insurance guidelines, 2015 - 'Ordonnance du DFI sur les prestations dans l'assurance obligatoire des soins en cas de maladie (Ordonnance sur les prestations de l'assurance des soins, OPAS)'(33) | Social health coverage guidelines | Swiss social health insurance coverage guidelines. | \ | Switzerland | information on what prenatal screening tests are covered under Swiss health care insurance (mandatory). | \ | \ | \ | A |
| The California prenatal screening programme (34) | Patient information booklet | \ | \ | California, USA | \ | \ | \ | \ | A |
| Torres-Aguilar et al., 2021(35) | Prospective pilot cohort study | To analyse the results of contingent screening, introducing cf-DNA testing into programme. | June 2017- June 2019 | Huelva, Spain (Andalucía health system) | The sensitivity, specificity, predictive values of conventional and cf-DNA screening calculated. Incidence of trisomies. | Andalucian public health system | No conflicts of interest | 16 | A+B |
| Van den Bogaert et al., 2021(36) | Retrospective cohort study | The performance of genome wide NIPT for both primary and secondary findings and the impact on practise and society following the first two years of national implementation. | 2017 - 2019 | Belgium | NIPT performance in general population, uptake of NIPT, detection of trisomies, false negatives, rare autosomal anomalies, Impact on the number of invasive tests; incidence of DS live births. | Partly funded by KULeven funding. | No conflicts of interest | 21 | A + B |
| Van der Meij, 2019 (37) | Prospective cohort study (implementation study) | To evaluate the first-year results of NIPT implementation as a first-tier screening tests for trisomies 21,18 and 13. Presenting the uptake and performance of NIPT. | April 2017 - April 2018 | The Netherlands | Test uptake; choice for reporting additional findings; failure rate; turn-around time (TAT, number of days between blood arrival at the NIPT laboratory and reporting in Periods); test performance of NIPT for trisomies 21, 18, and 13; and additional findings (rare autosomal trisomies [RATs] and SAs) | Grant from Netherlands Organisation for Health Research and Development | No conflicts of interest | 20 | A + B |
| Xue et al., 2019 (38) | Retrospective cohort study | Evaluate the results and validity of NIPT testing when compared to karyotype testing. | 2012-2017 | The Affiliated Suzhou Hospital of Nanjing Medical University | NIPT results, follow up results confirmed by invasive tests, performance of NIPT for each aneuploidy being tested. | Suzhou science and technology support programme. | No conflicts of interest | 18 | B |
| Zheng et al., 2022(39) | Retrospective single centre study | Evaluate the accuracy and feasibility of NIPT according to the results of NIPT and pregnancy outcomes | October 2014 - December 2020 | Xijing Hospital, China | delivery outcomes - survival, induced labour, abortion, karyotype analysis. | National key Research and Development Program of China | No conflicts of interest | 14 | B |
| Zhou et al., 2019 (40) | Prospective cohort study | Examine the factors affecting attitudes to prenatal diagnosis by analysing data of women after NIPT. | 2012-2017 | Changzhou maternity and child healthcare hospital - China | NIPT results and pregnancy outcomes for women positive after NIPT for trisomies. | National Natural Science Foundation of China grant | No conflicts of interest | 18 | B |
| Yuan et al., 2023(41) | Retrospective cohort study | Analyse the screening performance of NIPT and the differences in ultrasound phenotypes with abnormal NIPT results. | January 2018 – December 2021 | Xiangya Hospital of Central South University | Women who underwent NIPT enrolled, ultrasound follow-up performed throughout pregnancy, ultrasonographic findings divided up, those undergoing invasive testing and pregnancy outcomes. | Key research and development program of Hunan Province | No conflicts of interest | 14 | B |
| Walter et al., 2022(42) | Retrospective cohort study – single centre | To investigate the uptake of different factors of first trimester screening and the impact of invasive testing since the introduction of NIPT. | January to December 2019, retrospective data from 2012/13 | University Hospital Bonn (Germany) | Screening options following presentation for first trimester screening at the centre. Uptake of NIPT, risk group for Down’s syndrome, number undergoing invasive prenatal testing. | No funding received | No conflicts of interest | 12 | B |

*Table 1: Study characteristics of those included in part A of this systematic review. cfDNA = cell free DNA, another term for NIPT. *Gadsboll et al., is a review of surveys sent out to clinical experts in each available country, asking about the NIPT use in their country – from this we have taken the countries where clinicians said NIPT was used as part of a national or regional screening programme. These countries are included for the implementation of NIPT only. *Paper found through hand-searching. Where no data is available for the table, ‘\’ has been used.*

42 studies total

21 studies included in part A

31 studies included in part B

References

1. Akaishi R, Yamada T, Kawaguchi S, Kojima T, Koyama T, Umazume T, et al. Uptake of non-invasive prenatal testing by Japanese women. Vol. 45, Ultrasound in Obstetrics and Gynecology. 2015.

2. Bowden B, de Souza S, Puchades A, Williams K, Morgan S, Anderson S, et al. Implementation of non‐invasive prenatal testing within a national UK antenatal screening programme: Impact on women’s choices. Prenat Diagn [Internet]. 2022 May 24;42(5):549–56. Available from: https://onlinelibrary.wiley.com/doi/10.1002/pd.6131

3. Bjerregaard L, Stenbakken AB, Andersen CS, Kristensen L, Jensen CV, Skovbo P, et al. The rate of invasive testing for trisomy 21 is reduced after implementation of NIPT. Dan Med J. 2017;64(4).

4. Chen Y, Lu L, Zhang Y, Wang F, Ni Y, Wang Q, et al. Clinical application of expanded noninvasive prenatal testing for fetal chromosome abnormalities in a cohort of 39,580 pregnancies. Am J Med Genet A. 2022 May 1;188(5):1426–34.

5. Choe SA, Seol HJ, Kwon JY, Park CW, Kim M, Lee JY, et al. Clinical Practice Guidelines for Prenatal Aneuploidy Screening and Diagnostic Testing from Korean Society of Maternal-Fetal Medicine: (1) Prenatal Aneuploidy Screening. J Korean Med Sci. 2021 Jan 25;36(4).

6. Dap M, Caffin L, Perdriolle-Galet E, Bonnet C, Morel O. Is cell-free fetal DNA testing a safe option for women in a high-risk population after combined first-trimester testing? J Gynecol Obstet Hum Reprod. 2022;51(4).

7. Dougan SD, Okun N, Bellai-Dussault K, Meng L, Howley HE, Huang T, et al. Performance of a universal prenatal screening program incorporating cell-free fetal DNA analysis in Ontario, Canada. Can Med Assoc J. 2021 Aug 3;193(30):E1156–63.

8. Duvillier C, Dard R, Hervé B, Cohen C, Vialard F, Quibel T. Effects of the implementation of second-line prenatal cell-free DNA testing on termination of pregnancy in a French perinatal network. European Journal of Obstetrics and Gynecology and Reproductive Biology. 2021 Dec 1;267:36–41.

9. Flöck A, Tu NC, Rüland A, Holzgreve W, Gembruch U, Geipel A. Non-invasive prenatal testing (NIPT): Europe’s first multicenter post-market clinical follow-up study validating the quality in clinical routine. Arch Gynecol Obstet. 2017;296(5).

10. Gadsbøll K, Petersen OB, Gatinois V, Strange H, Jacobsson B, Wapner R, et al. Current use of noninvasive prenatal testing in Europe, Australia and the USA: A graphical presentation. Acta Obstet Gynecol Scand. 2020;99(6).

11. Garshasbi M, Wang Y, Hantoosh Zadeh S, Giti S, Piri S, Hekmat MR. Clinical Application of Cell-Free DNA Sequencing-Based Noninvasive Prenatal Testing for Trisomies 21, 18, 13 and Sex Chromosome Aneuploidy in a Mixed-Risk Population in Iran. Fetal Diagn Ther. 2020;47(3).

12. Yukon - Congenital Anomalies Support Yukon. An Aid to Decision-Making for Prenatal Screening. 2019 Feb.

13. Hill M, Barrett A, Choolani M, Lewis C, Fisher J, Chitty LS. Has noninvasive prenatal testing impacted termination of pregnancy and live birth rates of infants with Down syndrome? Prenat Diagn. 2017;37(13).

14. Hsiao CH, Chen CH, Cheng PJ, Shaw SW, Chu WC, Chen RC. The impact of prenatal screening tests on prenatal diagnosis in Taiwan from 2006 to 2019: a regional cohort study. BMC Pregnancy Childbirth. 2022 Dec 1;22(1).

15. Kou K, Poon C, Kwok S, Chan KY, Tang MH, Kan AS, et al. Effect of non-invasive prenatal testing as a contingent approach on the indications for invasive prenatal diagnosis and prenatal detection rate of Down’s syndrome. Hong Kong Medical Journal. 2016 May 6;

16. Kudryavtseva KEV, Kanivets KIV, Kievskaya KYuK, Baranov BII, Kovalev KVV, Korostelev KSA. Noninvasive prenatal testing in Russia: a population study. Akush Ginekol (Mosk). 2019 Dec 30;12_2019:28–33.

17. Lindquist A, Hui L, Poulton A, Kluckow E, Hutchinson B, Pertile MD, et al. State-wide utilization and performance of traditional and cell-free DNA-based prenatal testing pathways: the Victorian Perinatal Record Linkage (PeRL) study. Ultrasound in Obstetrics and Gynecology. 2020 Aug 1;56(2):215–24.

18. Liu Y, Jing X, Xing L, Liu S, Liu J, Cheng J, et al. Noninvasive Prenatal Screening Based on Second-Trimester Ultrasonographic Soft Markers in Low-Risk Pregnant Women. Front Genet. 2021 Dec 23;12.

19. Lund ICB, Petersen OB, Becher NH, Lildballe DL, Jørgensen FS, Ambye L, et al. National data on the early clinical use of non‐invasive prenatal testing in public and private healthcare in Denmark 2013–2017. Acta Obstet Gynecol Scand [Internet]. 2021 May 24;100(5):884–92. Available from: https://onlinelibrary.wiley.com/doi/10.1111/aogs.14052

20. Manegold-Brauer G, Bellin AK, Hahn S, De Geyter C, Buechel J, Hoesli I, et al. A new era in prenatal care: Non-invasive prenatal testing in Switzerland. Swiss Med Wkly. 2014;144.

21. Manotaya S, Xu H, Uerpairojkit B, Chen F, Charoenvidhya D, Liu H, et al. Clinical experience from Thailand: Noninvasive prenatal testing as screening tests for trisomies 21, 18 and 13 in 4736 pregnancies. Prenat Diagn. 2016;36(3).

22. Martínez-Payo C, Bada-Bosch I, Martínez-Moya M, Pérez-Medina T. Clinical results after the implementation of cell-free fetal DNA detection in maternal plasma. Journal of Obstetrics and Gynaecology Research. 2018;44(8).

23. McLennan A, Palma-Dias R, Da Silva Costa F, Meagher S, Nisbet DL, Scott F. Noninvasive prenatal testing in routine clinical practice - An audit of NIPT and combined first-trimester screening in an unselected Australian population. Australian and New Zealand Journal of Obstetrics and Gynaecology. 2016;56(1).

24. Norwegian Health Directorate. https://www.helsenorge.no/en/undersokelse-og-behandling/fetal-diagnostics/#nipt-for-trisomy-13-18-and-21. 2022. Prenatal diagnostics - examination of the fetus.

25. Olenev AS, Baranova EE, Sagaydak O V., Galaktionova AM, Kuznetsova ES, Kaplanova MT, et al. Adoption of a non-invasive prenatal test (NIPT) in prenatal screening in Moscow: First results. Russian Open Medical Journal. 2021;10(1).

26. Public Health England. Down’s syndrome, Edwards’ syndrome and  Patau’s syndrome: options after a higher  chance screening result. 2021.

27. Sainz JA, Torres MR, Peral I, Granell R, Vargas M, Carrasco P, et al. Clinical and Economic Evaluation after Adopting Contingent Cell-Free DNA Screening for Fetal Trisomies in South Spain. Fetal Diagn Ther. 2020;47(10).

28. Samura O, Sekizawa A, Suzumori N, Sasaki A, Wada S, Hamanoue H, et al. Current status of non-invasive prenatal testing in Japan. J Obstet Gynaecol Res. 2017 Aug;43(8):1245–55.

29. Sasaki Y, Yamada T, Tanaka S, Sekizawa A, Hirose T, Suzumori N, et al. Evaluation of the clinical performance of noninvasive prenatal testing at a Japanese laboratory. Journal of Obstetrics and Gynaecology Research. 2021;47(10).

30. Dr Gregor Smith. IMPORTANT CHANGES TO THE SCOTTISH PREGNANCY SCREENING PROGRAMME. 2020 Aug.

31. Ultra ARG interdisciplinary. Analysis of foetal DNA in the woman’s blood: non-invasive prenatal testing (NIPT) for trisomy 13, 18 and 21. 2016.

32. Shah F, French K, Osann K, Bocian M, Jones M, Korty L. Impact of Cell-Free Fetal DNA Screening on Patients’ Choice of Invasive Procedures after a Positive California Prenatal Screen Result. J Clin Med. 2014;3(3).

33. Ordinance on healthcare insurance benefits O. Swiss public health insurance guidelines  - “Ordonnance du DFI sur les prestations dans l’assurance obligatoire des soins en cas de maladie (Ordonnance sur les prestations de l’assurance des soins, OPAS).” 2015.

34. California Department of Public Health. California Prenatal Screening Program Patient Booklet . California ; 2022 Sep.

35. Torres Aguilar MR, Carrasco Salas P, Santos Rosa C, Bueno Rodríguez G, Martínez-Bonet E, Carreto Alba P, et al. Contingent prenatal screening for frequent aneuploidies with cell-free fetal DNA analysis. Taiwan J Obstet Gynecol. 2021;60(4).

36. Van Den Bogaert K, Lannoo L, Brison N, Gatinois V, Baetens M, Blaumeiser B, et al. Outcome of publicly funded nationwide first-tier noninvasive prenatal screening. Genetics in Medicine [Internet]. 2021 Jun;23(6):1137–42. Available from: https://linkinghub.elsevier.com/retrieve/pii/S109836002105214X

37. van der Meij KRM, Sistermans EA, Macville MVE, Stevens SJC, Bax CJ, Bekker MN, et al. TRIDENT-2: National Implementation of Genome-wide Non-invasive Prenatal Testing as a First-Tier Screening Test in the Netherlands. Am J Hum Genet. 2019;105(6).

38. Xue Y, Zhao G, Li H, Zhang Q, Lu J, Yu B, et al. Non-invasive prenatal testing to detect chromosome aneuploidies in 57,204 pregnancies. Mol Cytogenet. 2019;12(1).

39. Zheng Y, Li J, Zhang J, Yang H. The accuracy and feasibility of noninvasive prenatal testing in a consecutive series of 20,626 pregnancies with different clinical characteristics. J Clin Lab Anal. 2022;36(10).

40. Zhou Q, Zhu ZP, Zhang B, Yu B, Cai ZM, Yuan P. Clinical features and pregnancy outcomes of women with abnormal cell-free fetal DNA test results. Ann Transl Med. 2019;7(14).

41. Yuan X, Yong W, Dai L, Wang W, Wu L. The role of non-invasive prenatal testing and ultrasound in prenatal screening of fetal chromosomal abnormalities in singleton: a retrospective study. Ann Transl Med. 2023;11(2).

42. Walter A, Simonini C, Gembruch U, Flöck A, Strizek B, Geipel A. First Trimester Screening - Current Status and Future Prospects After Introduction of Non-invasive Prenatal Testing (NIPT) at a Tertiary Referral Center. Geburtshilfe Frauenheilkd. 2022 Oct;82(10):1068–73.

**2. Search strategy**

| Electronic databases searched (from inception to May 2023) | |
| --- | --- |
| Embase and MEDLINE | 1.Non-inavsive prenatal testing.mp. [mp=title, abstract, heading word, drug trade name, original title, device manufacturer, drug manufacturer, device trade name, keyword heading word, floating subheading word, candidate term word]    2.limit 1 to yr="2010 -Current"    3.non-invasive prenatal testing.mp. [mp=title, abstract, heading word, drug trade name, original title, device manufacturer, drug manufacturer, device trade name, keyword heading word, floating subheading word, candidate term word]    4.NIPT.mp. [mp=title, abstract, heading word, drug trade name, original title, device manufacturer, drug manufacturer, device trade name, keyword heading word, floating subheading word, candidate term word]    5.second-line prenatal screening.mp. [mp=title, abstract, heading word, drug trade name, original title, device manufacturer, drug manufacturer, device trade name, keyword heading word, floating subheading word, candidate term word]    6.second line pre-natal screening.mp. [mp=title, abstract, heading word, drug trade name, original title, device manufacturer, drug manufacturer, device trade name, keyword heading word, floating subheading word, candidate term word]    7.antenatal screening.mp. [mp=title, abstract, heading word, drug trade name, original title, device manufacturer, drug manufacturer, device trade name, keyword heading word, floating subheading word, candidate term word]    8.pregnancy screening.mp. [mp=title, abstract, heading word, drug trade name, original title, device manufacturer, drug manufacturer, device trade name, keyword heading word, floating subheading word, candidate term word]    9.Down's syndrome.mp. [mp=title, abstract, heading word, drug trade name, original title, device manufacturer, drug manufacturer, device trade name, keyword heading word, floating subheading word, candidate term word]    10.Trisomy 21.mp. [mp=title, abstract, heading word, drug trade name, original title, device manufacturer, drug manufacturer, device trade name, keyword heading word, floating subheading word, candidate term word]    11.chromosomal aneuploidy.mp. [mp=title, abstract, heading word, drug trade name, original title, device manufacturer, drug manufacturer, device trade name, keyword heading word, floating subheading word, candidate term word]    12.health care implementation.mp. [mp=title, abstract, heading word, drug trade name, original title, device manufacturer, drug manufacturer, device trade name, keyword heading word, floating subheading word, candidate term word]    13.service delivery.mp. [mp=title, abstract, heading word, drug trade name, original title, device manufacturer, drug manufacturer, device trade name, keyword heading word, floating subheading word, candidate term word]    14.implementation.mp. [mp=title, abstract, heading word, drug trade name, original title, device manufacturer, drug manufacturer, device trade name, keyword heading word, floating subheading word, candidate term word]    15.national screening programme.mp. [mp=title, abstract, heading word, drug trade name, original title, device manufacturer, drug manufacturer, device trade name, keyword heading word, floating subheading word, candidate term word]    16.uptake.mp. [mp=title, abstract, heading word, drug trade name, original title, device manufacturer, drug manufacturer, device trade name, keyword heading word, floating subheading word, candidate term word]    17.access.mp. [mp=title, abstract, heading word, drug trade name, original title, device manufacturer, drug manufacturer, device trade name, keyword heading word, floating subheading word, candidate term word]    18.pregnancy outcomes.mp. [mp=title, abstract, heading word, drug trade name, original title, device manufacturer, drug manufacturer, device trade name, keyword heading word, floating subheading word, candidate term word]    19.termination rate.mp. [mp=title, abstract, heading word, drug trade name, original title, device manufacturer, drug manufacturer, device trade name, keyword heading word, floating subheading word, candidate term word]    20.birth rate.mp. [mp=title, abstract, heading word, drug trade name, original title, device manufacturer, drug manufacturer, device trade name, keyword heading word, floating subheading word, candidate term word]    21.impact.mp. [mp=title, abstract, heading word, drug trade name, original title, device manufacturer, drug manufacturer, device trade name, keyword heading word, floating subheading word, candidate term word]    22. 12 or 13 or 14 or 15 or 16 or 17 or 18 or 19 or 20 or 21    23.9 or 10 or 11    24.3 or 4 or 7 or 8    25. 22 and 23 and 24 |
| CINAHL | [Noninvasive prenatal testing OR NIPT ] AND [down’s syndrome OR trisomy 21 OR down syndrome ] AND [antenatal screening OR prenatal screening ] AND [implementation OR implementing OR facilitating ] |
| Scopus | title  =  ( "noninvasive prenatal screening"  OR  "NIPT"  OR  "noninvasive prenatal testing"  OR  "cell-free DNA" )  AND  ( "Down$ syndrome"  OR  "Trisom$ 21"  OR  "T21"  OR  "downs" ) |
