# Supplementary material for: The implementation and impact of non-invasive prenatal testing (NIPT) for Down’s syndrome into antenatal screening programmes: A systematic review and meta-analysis
Source: PLoS One. 2024 May 16;19(5):e0298643. doi: 10.1371/journal.pone.0298643 (PMC11098470; doi:10.1371/journal.pone.0298643)
Supplement: S2 Table — (DOCX) [file pone.0298643.s003.docx]

**S2 Table: Termination of pregnancy laws in part A included countries/autonomous regions.**

| **Country / state / province** | **Termination of pregnancy (TOP) law** | | **Reference** |
| --- | --- | --- | --- |
|  | **Legal/restricted** | **Gestational limits for TOP** |  |
| Hong Kong | Legal: 1. If continuing pregnancy would risk the pregnant woman’s mental or physical health 2. If the baby will be born with a severe disability | 24 weeks | Family Health Service, Department of Health, The Government of Hong Kong Special Administrative Region (1) |
| Japan | Legal: social and economic grounds (spousal authorisation required) | No limit stated | * |
| Victoria, Australia | Legal: on request | 24 weeks  (beyond 24 weeks must be approved by two medical practitioners) | * |
| Switzerland | Legal: on request | 12 weeks | * |
| Taiwan | Legal: social or economic grounds (required: spousal authorisation, permitted for foetal diagnosis) | Not reported | * |
| Ontario, Canada | Legal: on request | 24 weeks | National Abortion Federation, Canada (2) |
| Sweden | Legal: on request | 18 weeks | * |
| Belgium | Legal: on request | 14 weeks | * |
| Denmark | Legal: on request | 12 weeks | * |
| South Spain – Andalucía | Legal: on request | 14 weeks  (22 weeks in cases of risk to maternal life or fetal disability) | * |
| The Netherlands | Legal: on request | No limit stated | * |
| Yukon, Canada | Legal: on request | 12 weeks | National Abortion Federation, Canada |
| Wales, UK | Legal: broad social and economic grounds | 24 weeks  (No gestational limit in cases of risk to maternal life or fetal disability) | * |
| Poissy Saint-Germain, France | Legal: on request | 16 weeks | * |
| Poland | Restricted: only cases of rape, incest, or to preserve maternal life. | N/A | * |
| Romania | Legal: on request | 14 weeks | * |
| Iceland | Legal: on request | 22 weeks | * |
| Lithuania | Legal: on request | 12 weeks | * |
| Italy | Legal: on request | 90 days | * |
| Finland | Legal: on requestion | 12 weeks | * |
| Slovenia | Legal: on request | 12 weeks | * |
| USA | Restricted: varies by state (as of June 2022) abortion is now regulated at the state level and has been heavily restricted in states across the U.S e.g., very early (6 week) gestational limits on TOP or prohibited except when ‘necessary to preserve life’. | Where legal, varies by state | * |
| Korea | Legal: on request | No limit stated | * |
| Moscow, Russia | Legal: on request | No limit stated | * |
| Scotland, UK | Legal: broad social and economic grounds | 24 weeks  (No gestational limit in cases of risk to maternal life or fetal disability) | * |
| England, UK | Legal: broad social and economic grounds | 24 weeks  (No gestational limit in cases of risk to maternal life or fetal disability) | * |
| California, USA | Legal: on request | No limit stated | * |
| Norway | Legal: on request | 12 weeks | * |

Table to describe the legal guidelines around termination of pregnancy in each of the included countries/autonomous regions included in part A. The laws have been categorised into ‘legal’, ‘restricted’ or ‘illegal’, with further information about the access to legal/restricted terminations of pregnancy in that population. * Information sourced from the Centre for Reproductive Rights World Abortion Laws map – accessed from The World's Abortion Laws - Center for Reproductive Rights (3)

References

1. Woman Health - Termination of pregnancy [Internet]. Department of Health - Hong Kong ; [cited 2023 Dec 18]. Available from: https://www.fhs.gov.hk/english/health_info/woman/15673.html#:~:text=In%20Hong%20Kong%2C%20termination%20of%20pregnancy%20is%20legal,as%20a%20result%20of%20physical%20or%20mental%20abnormality.
2. Abortion coverage by region [Internet]. [cited 2023 Dec 18]. Available from: https://nafcanada.org/abortion-coverage-region/
3. The world’s abortion laws [Internet]. 2023 [cited 2023 Dec 18]. Available from: https://reproductiverights.org/maps/worlds-abortion-laws
