# Supplementary material for: The implementation and impact of non-invasive prenatal testing (NIPT) for Down’s syndrome into antenatal screening programmes: A systematic review and meta-analysis
Source: PLoS One. 2024 May 16;19(5):e0298643. doi: 10.1371/journal.pone.0298643 (PMC11098470; doi:10.1371/journal.pone.0298643)
Supplement: S1 File — Systematic review protocol document, registered on the PROSPERO database. (PDF) [file pone.0298643.s004.pdf]

National Institute for Health Research

University of York
Centre for Reviews and Dissemination

Systematic review

A list of fields that can be edited in an update can be found here

1. * Review title.

Give the title of the review in English

A systematic review of the implementation and impact of non-invasive prenatal testing (NIPT) for Down's syndrome in national antenatal screening programmes

2. Original language title.

For reviews in languages other than English, give the title in the original language. This will be displayed with the English language title.

3. * Anticipated or actual start date.

Give the date the systematic review started or is expected to start.

18/03/2022

4. * Anticipated completion date. [2 changes]

Give the date by which the review is expected to be completed.

01/10/2023

5. * Stage of review at time of this submission. [2 changes]

This field uses answers to initial screening questions. It cannot be edited until after registration.

Tick the boxes to show which review tasks have been started and which have been completed.

Update this field each time any amendments are made to a published record.

The review has not yet started: No

Review stage	Started	Completed
Preliminary searches	Yes	Yes
Piloting of the study selection process	Yes	Yes
Formal screening of search results against eligibility criteria	Yes	Yes
Data extraction	Yes	Yes
Risk of bias (quality) assessment	Yes	Yes
Data analysis	Yes	Yes

Provide any other relevant information about the stage of the review here.

6. * Named contact.

The named contact is the guarantor for the accuracy of the information in the register record. This may be any member of the review team.

Elinor Sebire

Email salutation (e.g. "Dr Smith" or "Joanne") for correspondence:

Ms Sebire

7. * Named contact email.

Give the electronic email address of the named contact.

e.sebire.21@abdn.ac.uk

8. Named contact address

Give the full institutional/organisational postal address for the named contact.

Polwarth Building, University of Aberdeen, Aberdeen, AB25 2ZD

9. Named contact phone number.

Give the telephone number for the named contact, including international dialling code.

10. * Organisational affiliation of the review.

Full title of the organisational affiliations for this review and website address if available. This field may be completed as 'None' if the review is not affiliated to any organisation.

University of Aberdeen

Organisation web address:

https://www.abdn.ac.uk/

11. * Review team members and their organisational affiliations. [1 change]

Give the personal details and the organisational affiliations of each member of the review team. Affiliation refers to groups or organisations to which review team members belong. **NOTE: email and country now MUST be entered for each person, unless you are amending a published record.**

Ms Elinor Sebire. University of Aberdeen
Dr Rute Vieira. University of Aberdeen
Dr Chithramali Rodrigo. University of Aberdeen

12. * Funding sources/sponsors. [1 change]

Details of the individuals, organizations, groups, companies or other legal entities who have funded or sponsored the review.

Medical Research Scotland Studentship - funds the overall programme of work completed as part of PhD.

No specific funding for this review.

Grant number(s)

State the funder, grant or award number and the date of award

MRS Studentship, award date October 2021

13. * Conflicts of interest.

List actual or perceived conflicts of interest (financial or academic).

None

14. Collaborators. [1 change]

Give the name and affiliation of any individuals or organisations who are working on the review but who are not listed as review team members. **NOTE: email and country must be completed for each person, unless you are amending a published record.**

Dr Sohinee Bhattacharya. University of Aberdeen
Dr Mairead Black. University of Aberdeen
Dr Rachael Wood. Public Health Scotland

15. * Review question. [1 change]

State the review question(s) clearly and precisely. It may be appropriate to break very broad questions down into a series of related more specific questions. Questions may be framed or refined using PI(E)COS or similar where relevant.

How has non-invasive prenatal testing (NIPT) for Down’s syndrome (DS) been implemented as an antenatal screening test globally?

Has the implementation of NIPT affected the uptake (n/%) of screening for DS among pregnant populations?

To what extent has NIPT impacted the prevalence of adverse pregnancy or live birth outcomes for babies with DS?

16. * Searches. [1 change]

State the sources that will be searched (e.g. Medline). Give the search dates, and any restrictions (e.g. language or publication date). Do NOT enter the full search strategy (it may be provided as a link or attachment below.)

Databases to be searched: MEDLINE, EMBASE, CINAHL, Scopus

Search will be adapted for Web of Science and Google Scholar.

Internet resources: search engine (Google) will also be used to hand search for any relevant government/healthcare statistics relating to countries and populations already identified and included after the systematic search.

No restriction on publication date or language.

The reference lists of all eligible studies will also be checked="checked" value="1" for any relevant papers not yet included.

17. URL to search strategy. [1 change]

Upload a file with your search strategy, or an example of a search strategy for a specific database, (including the keywords) in pdf or word format. In doing so you are consenting to the file being made publicly accessible. Or provide a URL or link to the strategy. Do NOT provide links to your search **results**.

Alternatively, upload your search strategy to CRD in pdf format. Please note that by doing so you are consenting to the file being made publicly accessible.

Do not make this file publicly available until the review is complete

18. * Condition or domain being studied.

Give a short description of the disease, condition or healthcare domain being studied in your systematic review.

Down's syndrome (trisomy 21).

19. * Participants/population.

Specify the participants or populations being studied in the review. The preferred format includes details of both inclusion and exclusion criteria.

Pregnant populations in any country / state offered NIPT for the prenatal screening of Down’s syndrome.

20. * Intervention(s), exposure(s).

Give full and clear descriptions or definitions of the interventions or the exposures to be reviewed. The preferred format includes details of both inclusion and exclusion criteria.

Implementation of NIPT as part of a state screening.

Definitions: antenatal screening is any test performed before birth that is not diagnostic (offers a chance of risk for a particular condition). Non-invasive prenatal testing (NIPT) is a screening test that uses a maternal blood sample to access cell-free DNA originating from the foetus. This can give a risk ratio for the chance of the baby having DS.

Inclusion/exclusion criteria: only studies including NIPT offered for DS and as a screening test will be included. NIPT can be offered in the private or public sector.

21. * Comparator(s)/control.

[1 change]

Where relevant, give details of the alternatives against which the intervention/exposure will be compared (e.g. another intervention or a non-exposed control group). The preferred format includes details of both inclusion and exclusion criteria.

The implementation and impact of NIPT will be compared, where data exists for the same country/state, against outcomes of interest for traditional first-line (biochemical) screening, invasive prenatal testing (amniocentesis or chorionic villus sampling), i.e. outcomes of interest from antenatal screening pre-NIPT implementation. This will be sought by hand-searching databases and search engines for any relevant publications.

22. * Types of study to be included.

Give details of the study designs (e.g. RCT) that are eligible for inclusion in the review. The preferred format includes both inclusion and exclusion criteria. If there are no restrictions on the types of study, this should be stated.

As this is a review of an implemented healthcare service, experimental designs, e.g., randomised control trials are not appropriate for inclusion.

Included - observational studies: cohort, cross sectional, case-control; all published, official government or healthcare provision statistics.

23. Context.

[1 change]

Give summary details of the setting or other relevant characteristics, which help define the inclusion or exclusion criteria.

Non-invasive prenatal testing (NIPT) was first used in clinical practise for pre-natal screening in 2011, and since then has been adopted by many healthcare systems globally to screen for various genetic conditions. NIPT analyses genetic material originating from the placenta which circulates in the maternal blood stream to screen for certain chromosomal aneuploidies, most commonly trisomies 13, 18 and 21. Of these trisomies, Down's syndrome (trisomy 21, DS) has the highest population prevalence in Europe.

As this is a dynamic field, with an increasing number of countries adopting NIPT into their antenatal screening programmes, an updated systematic review is necessary to understand the impact globally of the implementation of this new technology on the pregnancy outcomes and births of those with DS. Previous reviews have addressed uptake as a proportion of the pregnant population, but other countries have since implemented and currently there is no systematic review that also looks to address the comparative impact of introducing NIPT on babies with DS.

NIPT for DS was introduced into the Scottish National Health Service antenatal screening programme in September 2020. In order to evaluate the impact of this new screening procedure, it is important to understand how it has been implemented in other countries.

Thus, this systematic review will seek to evaluate how NIPT has been introduced into the antenatal screening programmes of other countries globally, examine the uptake of this procedure and discuss how this may influence the live birth rate and outcomes of babies born with DS.

24. * Main outcome(s).

[1 change]

Give the pre-specified main (most important) outcomes of the review, including details of how the outcome is defined and measured and when these measurement are made, if these are part of the review inclusion criteria.

1. When is NIPT offered and to whom is NIPT offered in a population?

- Implementation of screening pathway (1st/2nd line/other)?

- Risk threshold for eligibility? [e.g. 1:150];

- Is NIPT publicly or privately funded?

- Other maternal characteristics for eligibility: age, family history of DS, multiplicity, etc.;

- Any specific exclusions from being offered NIPT?

2. Uptake: how many eligible individuals opt for NIPT?

- Number who opt for NIPT out of total eligible population (n/%);

- Age (or age groups) opting for NIPT (years).

3. Impact: live births, spontaneous foetal loss, and termination rates of babies with DS after NIPT.

- Termination rate after NIPT (n/%);

- Spontaneous loss rate after NIPT (n/%);

- Live birth rate with DS after NIPT (n/%);

- Any other reported pregnancy / live birth outcomes relating to babies with DS e.g. detection rate of DS in the population, incidence of invasive prenatal diagnosis procedures.

4. Control: pre-NIPT screening trends for DS (in country/state where NIPT available).

- Availability of DS screening pre-NIPT (private/public access, eligibility criteria) in country of interest;

- Pre-NIPT uptake of DS screening (n/%).

- Pre-NIPT impact data (as above): including live birth rate, spontaneous loss rate and termination rate after screening (n/%) for pregnancies and births with DS.

Measures of effect

Please specify the effect measure(s) for you main outcome(s) e.g. relative risks, odds ratios, risk difference, and/or 'number needed to treat.

25. * Additional outcome(s). [1 change]

List the pre-specified additional outcomes of the review, with a similar level of detail to that required for main outcomes. Where there are no additional outcomes please state ‘None’ or ‘Not applicable’ as appropriate to the review

None.

Measures of effect

Please specify the effect measure(s) for you additional outcome(s) e.g. relative risks, odds ratios, risk difference, and/or 'number needed to treat.

26. * Data extraction (selection and coding). [1. change]

Describe how studies will be selected for inclusion. State what data will be extracted or obtained. State how this will be done and recorded.

Study selection: two independent reviewers will undertake screening and data extraction phases (second reviewer undertake at least 10% of screening). Title and abstract screening will be followed by full text screening using reference software Rayyan, with any discrepancies discussed between the reviewers. Full text screening form will be used to justify each inclusion or exclusion decision. Reference lists of included papers will also be checked="checked" value="1" for relevant papers. All excluded reviews after the full-text phase will be listed in a ‘characteristics of excluded studies’ table. The screening and selection process will be reported using the PRISMA flow chart.

Data extraction: two independent review authors will extract data from included studies. A data extraction form will be developed and trialled on at least 2 papers before commencing data extraction. Data to be extracted will include study characteristics (design, aims, description of study cohort (n, age), country, funding source and declaration of interest) and outcome measures listed above. The form will be divided into part A (outcomes 1 and 2) and part B (outcomes 3 and 4). Collected study characteristics will be presented in a ‘Characteristics of included studies’ table. Data will be entered into a central table and checked="checked" value="1" for accuracy against the data extraction forms by the second reviewer independently.

Any disputes that do not reach agreement will be taken to review collaborators listed for their final decision. Collaborators will also help to test and approve any screening and data extraction forms.

27. * Risk of bias (quality) assessment. [1. change]

State which characteristics of the studies will be assessed and/or any formal risk of bias/quality assessment tools that will be used.

The Down's and black quality checklist for both randomised and non-randomised studies will be used for quality assessment. Two review authors will independently assess the quality of the evidence for 10% of the papers and judge agreement, then the primary reviewer will complete the rest of the quality assessment. This will be considered in the study summary tables and critical appraisal of each included study.

28. * Strategy for data synthesis. [1. change]

Describe the methods you plan to use to synthesise data. This **must not be generic text** but should be **specific to your review** and describe how the proposed approach will be applied to your data. If meta-analysis is planned, describe the models to be used, methods to explore statistical heterogeneity, and software package to be used.

Appropriate graphical and descriptive analysis will be used to summarise and visually compare outcome measures between countries. Narrative synthesis will be used where data between publications is not comparable. If sufficient data is available to ensure meaningful conclusions and where studies are found to be similar enough to allow pooling of the data (comparable population size, implementation of NIPT, outcome measures and an available control group) then a random effects meta-analysis model will be used to produce pooled effect sizes for outcomes of interest (using RevMan 5.4 software). Meta-analysis results will be visualised using forest plots.

We will explore the comparison in implementation and uptake of NIPT, and its effects on pregnancy and live birth outcomes with DS in each population.

if possible, subgroup analysis based on economic status of the country or the types of NIPT implementation will be used to understand the impact of these factors on the impact of pregnancy and live birth outcomes.

Heterogeneity:

Heterogeneity will be quantified using the I^2 statistic (50% or more considered high levels of heterogeneity). Where heterogeneity is significant across included studies, we will use a random-effects meta-analysis which should control for some of the heterogeneity. A sensitivity analysis will also be used to meta-analyse subgroups of similar studies. Re-meta-analysing the data while removing one study at a time should demonstrate whether some studies are more influential than others. Narrative synthesis approach will be taken instead of meta-analysis where appropriate to do so.

21. * Analysis of subgroups or subsets. [1 change]

State any planned investigation of ‘subgroups’. Be clear and specific about which type of study or participant will be included in each group or covariate investigated. State the planned analytic approach.

The outcome measures and results are expected to vary greatly between countries, due to the huge variation in access and provision of healthcare services worldwide, as well as the influence of cultural and societal differences on the use of prenatal screening and terminations. We will explore possible reasons for variation by examining factors such as general access to healthcare in each population (e.g., access to insurance, public funding, eligibility criteria, economic status of the country), any religious or societal influences that might be prominent in the population and could affect the differential reporting of outcomes

(i.e. state religion, abortion laws). Studies with population similar characteristics mentioned will be grouped and meta-regression can be used to explore sources of heterogeneity from these factors collected from each study.

30. * Type and method of review.

Select the type of review, review method and health area from the lists below.

Type of review

Cost effectiveness
No

Diagnostic
No

Epidemiologic
No

Individual patient data (IPD) meta-analysis
No

Intervention
Yes

Living systematic review
No

Meta-analysis
Yes

Methodology
No

Narrative synthesis
Yes

Network meta-analysis
No

Pre-clinical
No

Prevention
No

Prognostic
No

Prospective meta-analysis (PMA)
No

Review of reviews

No

Service delivery

Yes

Synthesis of qualitative studies

No

Systematic review

Yes

Other

No

Health area of the review

Alcohol/substance misuse/abuse

No

Blood and immune system

No

Cancer

No

Cardiovascular

No

Care of the elderly

No

Child health

No

Complementary therapies

No

COVID-19

No

Crime and justice

No

Dental

No

Digestive system

No

Ear, nose and throat

No

Education

No

Endocrine and metabolic disorders
No

Eye disorders
No

General interest
No

Genetics
No

Health inequalities/health equity
No

Infections and infestations
No

International development
No

Mental health and behavioural conditions
No

Musculoskeletal
No

Neurological
No

Nursing
No

Obstetrics and gynaecology
No

Oral health
No

Palliative care
No

Perioperative care
No

Physiotherapy
No

Pregnancy and childbirth
Yes

Public health (including social determinants of health)

Yes

Rehabilitation

No

Respiratory disorders

No

Service delivery

Yes

Skin disorders

No

Social care

No

Surgery

No

Tropical Medicine

No

Urological

No

Wounds, injuries and accidents

No

Violence and abuse

No

31. Language.

Select each language individually to add it to the list below, use the bin icon to remove any added in error.

English

There is not an English language summary

32. * Country.

Select the country in which the review is being carried out. For multi-national collaborations select all the countries involved.

Scotland

33. Other registration details.

Name any other organisation where the systematic review title or protocol is registered (e.g. Campbell, or The Joanna Briggs Institute) together with any unique identification number assigned by them. If extracted data will be stored and made available through a repository such as the Systematic Review Data Repository (SRDR), details and a link should be included here. If none, leave blank.

34. Reference and/or URL for published protocol.

If the protocol for this review is published provide details (authors, title and journal details, preferably in Vancouver format)

Add web link to the published protocol.

Or, upload your published protocol here in pdf format. Note that the upload will be publicly accessible.

No I do not make this file publicly available until the review is complete

Please note that the information required in the PROSPERO registration form must be completed in full even if access to a protocol is given.

35. Dissemination plans.

Do you intend to publish the review on completion?

Yes

Give brief details of plans for communicating review findings.?

The systematic review will be submitted for publication to relevant academic journals. Abstracts to present review findings will be submitted to conferences of interest.

36. Keywords.

Give words or phrases that best describe the review. Separate keywords with a semicolon or new line. Keywords help PROSPERO users find your review (keywords do not appear in the public record but are included in searches). Be as specific and precise as possible. Avoid acronyms and abbreviations unless these are in wide use.

Down's syndrome

Trisomy 21

NIPT

Non-invasive prenatal testing

Prenatal screening pathway

Health service implementation

37. Details of any existing review of the same topic by the same authors.

If you are registering an update of an existing review give details of the earlier versions and include a full bibliographic reference, if available.

38. * Current review status. [1 change]

Update review status when the review is completed and when it is published.New registrations must be ongoing so this field is not editable for initial submission.

Please provide anticipated publication date

Review_Completed_not_published

39. Any additional information.

Provide any other information relevant to the registration of this review.

40. Details of final report/publication(s) or preprints if available.

Leave empty until publication details are available OR you have a link to a preprint (NOTE: this field is not editable for initial submission). List authors, title and journal details preferably in Vancouver format.

Give the link to the published review or preprint.
