# Supplementary material for: The implementation and impact of non-invasive prenatal testing (NIPT) for Down’s syndrome into antenatal screening programmes: A systematic review and meta-analysis
Source: PLoS One. 2024 May 16;19(5):e0298643. doi: 10.1371/journal.pone.0298643 (PMC11098470; doi:10.1371/journal.pone.0298643)
Supplement: S3 File — Details of subgroup/meta-regression data analysis in part B of the systematic review. (DOCX) [file pone.0298643.s006.docx]

# S3 File - Appendix 2

# Data analysis (part B)

This document details further statistical analyses mentioned in the main body of the systematic review. Full dataset used for part B is available by request from authors.

1. Data reported by studies with both pre and post NIPT implementation period data:
2. The proportion of higher chance women after biochemical screening (total) who went on to have an invasive prenatal diagnosis (IPD) for Down’s syndrome (DS) (events). These studies report outcomes before and after NIPT has been implemented into their antenatal screening pathways for DS. Proportion (%) opting for IPD calculated, and direction of change shown.

Table 1: Table displaying the raw data and proportion of IPDs in higher chance pregnancies after traditional screening in both the pre- and post- NIPT periods. % shown for each period, and direction of change between periods.

| **Study ID** | **Pre NIPT** | | **Post NIPT** | | **% pre NIPT** | **% post NIPT** | **Change?** |
| --- | --- | --- | --- | --- | --- | --- | --- |
|  | Events | Total | Events | Total |  |  |  |
| Bjerregaard et al., 2017 | 177 | 253 | 145 | 302 | 70 | 48 | ↓ |
| Kou et al., 2016** | 262 | 306 | 474 | 763 | 85.6 | 62 | ↓ |
| Manegold-Brauer et al., 2014 | 21 | 37 | 8 | 20 | 56.8 | 40 | ↓ |
| Martinez-Payo et al. 2018 | 235 | 253 | 19 | 55 | 92.9 | 35 | ↓ |
| Shah et al., 2014 | 117 | 243 | 72 | 243 | 48 | 30 | ↓ |
| **Pooled estimate total (random effects, 95% CI)** | | | | | **75% (53%, 88%)** | **43% (31%, 56%)** | **↓** |

Overall, we can see that there is a trend towards a reduction in the proportion of women who have a high chance result after traditional first line screening who go on to have an IPD test after NIPT has been introduced into the screening pathway.

N.B. This is only applicable to screening pathways where NIPT has been introduced as a second line screening test.

1. Meta-analysis output (forest plot) for the proportion of pregnant women opting for invasive prenatal diagnosis (IPD) after a high chance NIPT (offered first or second line):


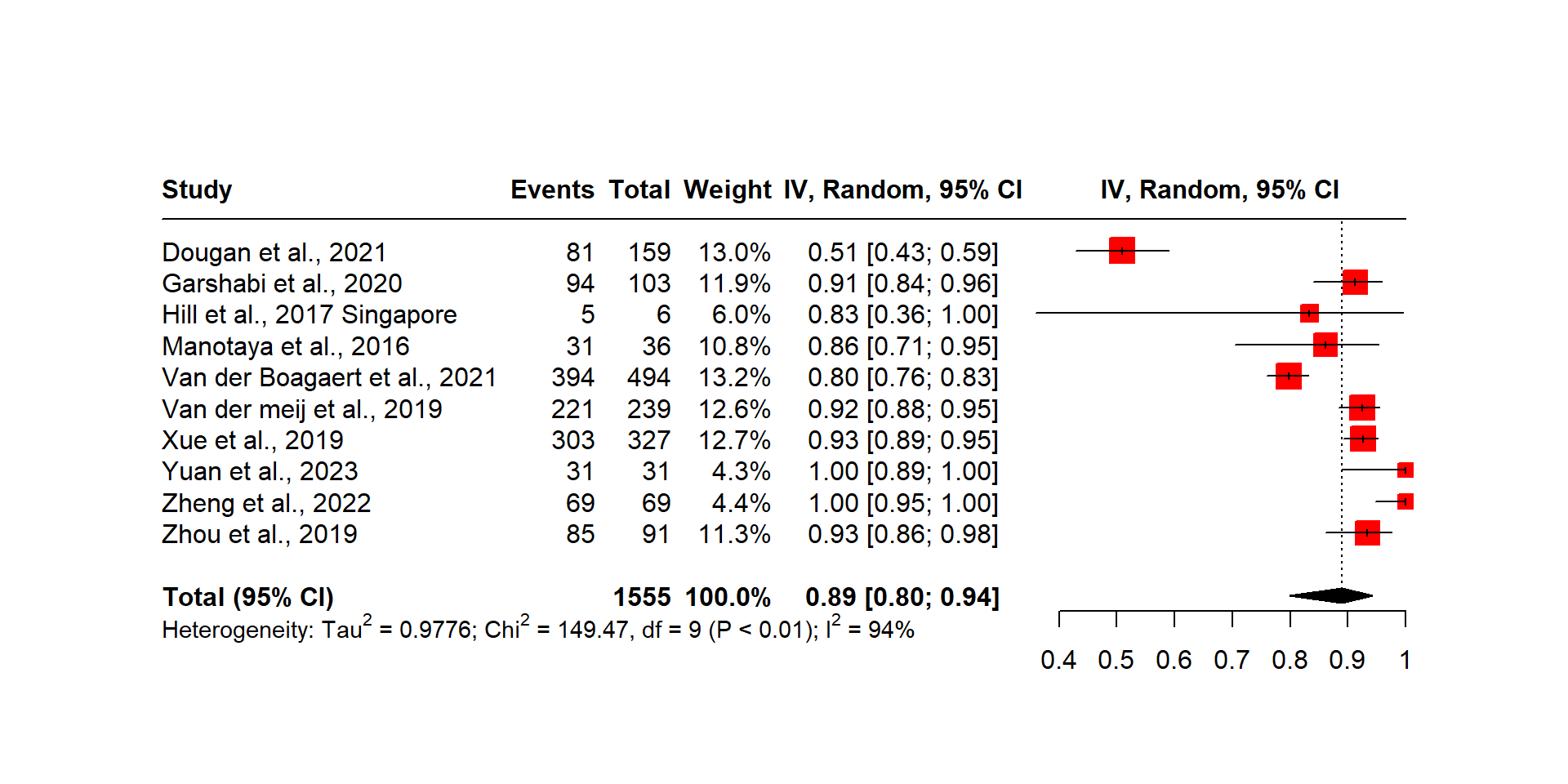


1. Sensitivity analyses

The outputs from sensitivity analysis, including forest plots are provided below. These analyses omitted one study at a time from the meta-analysis, to look for heterogeneity between the studies. Funnel plots for publication bias are also presented for each meta-analysis run.

1. Pre-NIPT sensitivity analysis (IPDs)

Sensitivity analysis to determine if there is a significant effect of removing each study from the random effects model – on the overall pooled proportion or the % heterogeneity.

The table below presents the output of the sensitivity analysis, also shown in the forest plot. Subsequent sensitivity analyses will only be presented using the forest plot.

Table 2: pre-NIPT period sensitivity analysis - omitting one study at a time.

| **Study omitted** | **Proportion (random effects model)** | **95% CI** | **Tau^2^** | **Tau** | **I^2^** |
| --- | --- | --- | --- | --- | --- |
| Bjerregaard et al., 2017 | 0.7576 | [0.4783; 0.9142] | 1.5137 | 1.2303 | 97.8% |
| Kou et al., 2016 | 0.7110 | [0.4386; 0.8857] | 1.3203 | 1.1490 | 96.9% |
| Manegold-Brauer et al., 2014 | 0.7807 | [0.5377; 0.9159] | 1.2726 | 1.1281 | 97.8% |
| Martinez-Payo et al. 2018 | 0.6719 | [0.4769; 0.8214] | 0.6406 | 0.8004 | 96.4% |
| Shah et al., 2014 | 0.7988 | [0.5996; 0.9132] | 0.9375 | 0.9683 | 94.6% |
|  |  |  |  |  |  |
| Total pooled estimate | 0.7464 | [0.5318; 0.8841] | 1.1350 | 1.0653 | 97.1% |

*Details on meta-analytical method: Inverse variance method, Restricted maximum-likelihood estimator for tau^2, Logit transformation*

Figure 1: Forest plot of sensitivity analysis for invasive prenatal diagnosis in the pre-NIPT period.


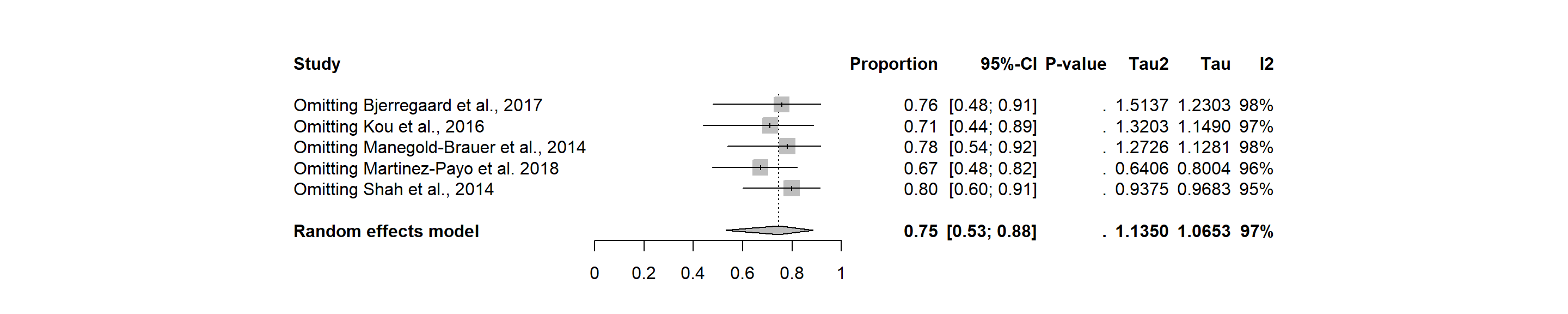
From this analysis, there does not seem to be a study that is particularly influential for the pooled estimate or heterogeneity (I^2^). Results show similar values for pooled estimates and heterogeneity, with overlapping CIs. Omitting Shah et al., reduced the heterogeneity by the most - 2%.


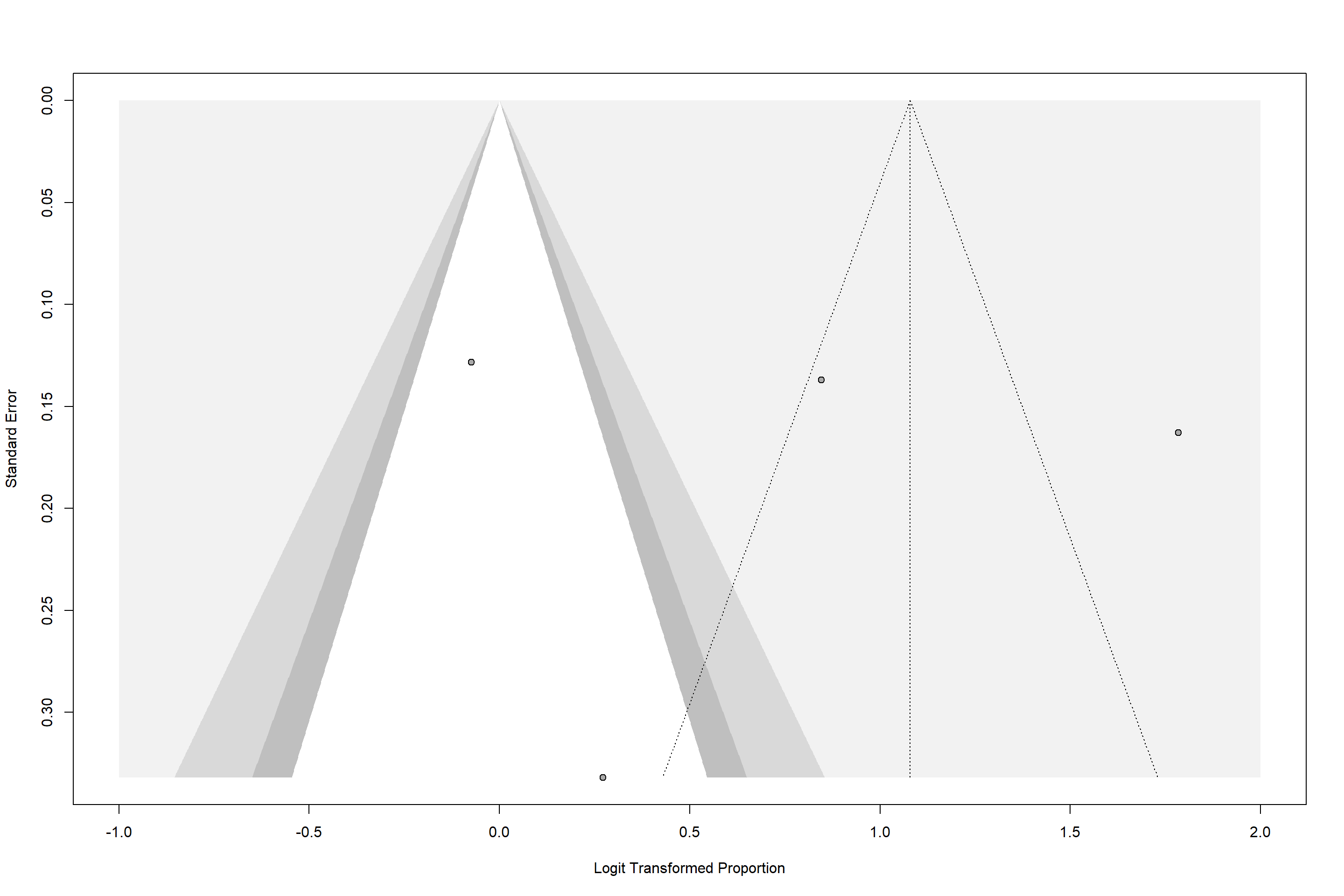


Funnel plot for publication bias of pre-NIPT data looking at IPDs (above). There are too few studies to interpret this plot. The layout of the SEs vs LT proportions is random.

Figure 2: Funnel plot

1. Post-NIPT sensitivity analysis (IPDs)
2. Sensitivity analysis for data on proportion undergoing IPDs in the post-NIPT implementation period (for those studies that also provided pre-NIPT data).


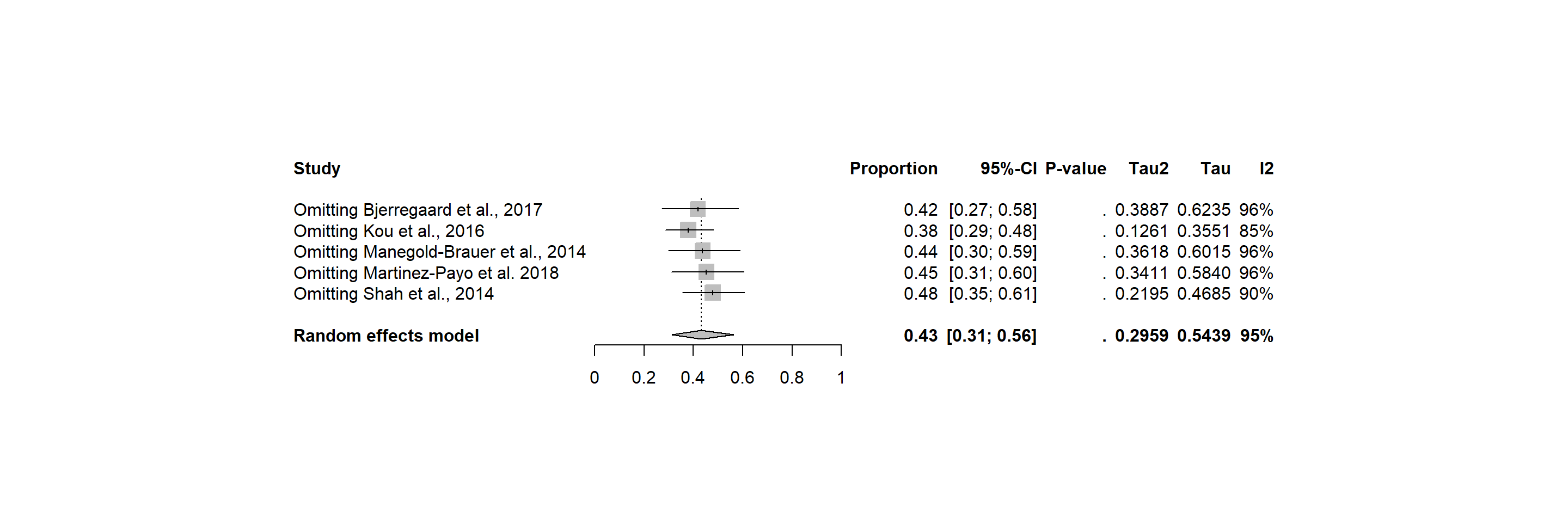
This analysis does not show one particular study as having a greater effect over the pooled estimate or heterogeneity. All similar values, with overlapping CIs. Omitting Kou et al reduced the heterogeneity the most, but CI still overlap, and this change doesn’t seem to be significant.

Figure 3: Sensitivity analysis forest plot - post NIPT


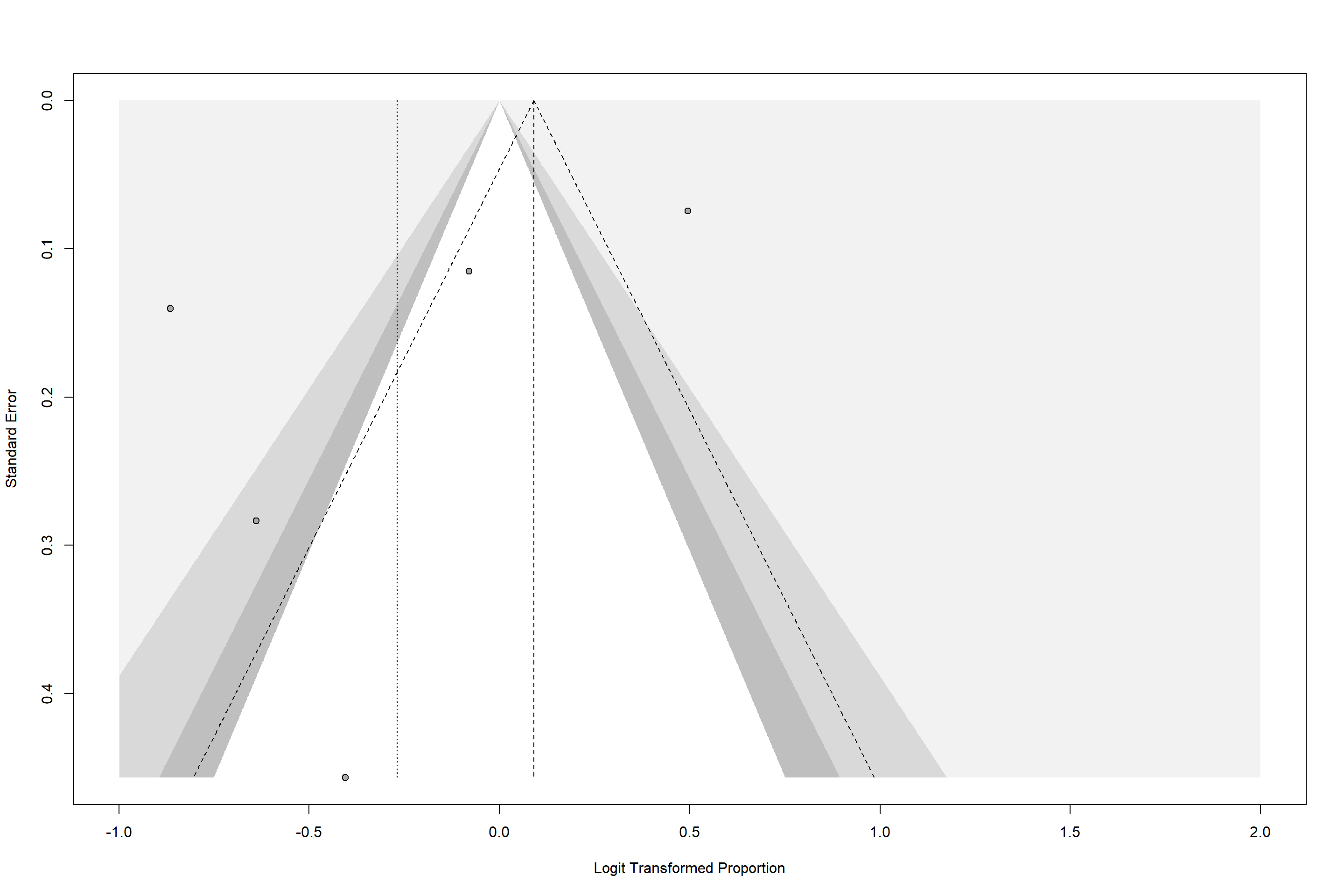
Funnel plot for the publication bias for this data:

Publication bias explored through funnel plot of the SE of the data. There is asymmetry in the plot, which may point to publication bias however with such few studies included in this data we are unable to infer much from this data spread.

Figure 4: Funnel plot, post NIPT

1. Post NIPT studies reporting IPDs after biochemical first line screening high chance result:


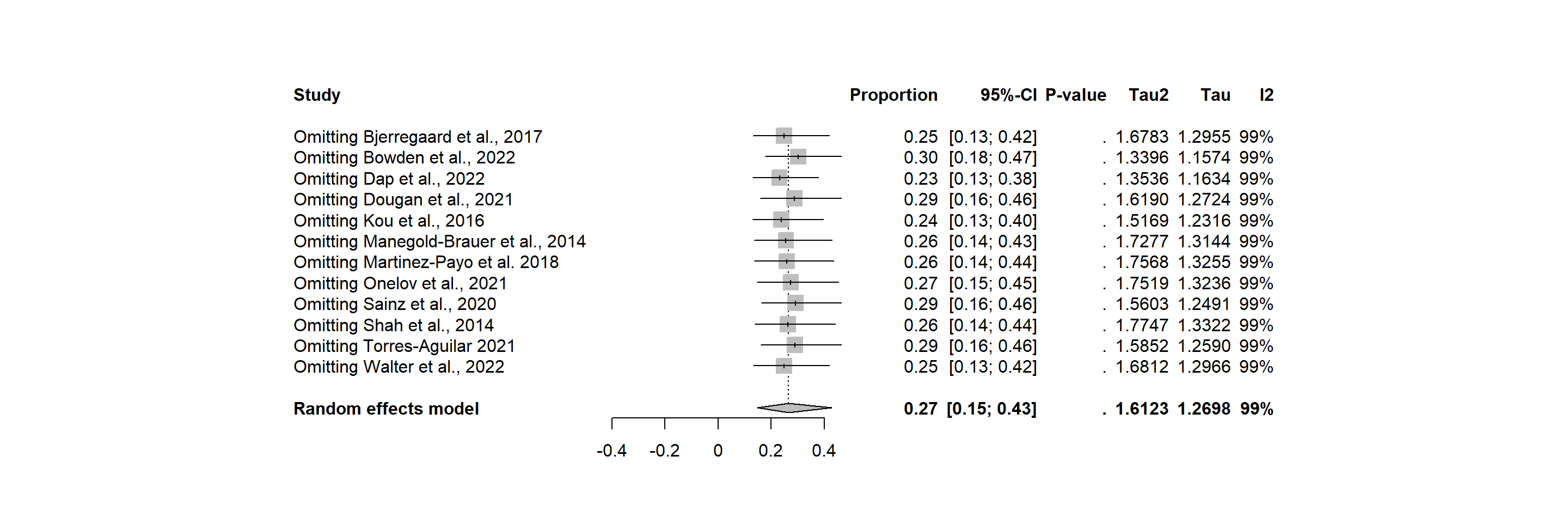
NIPT offered as a second line screening test, after biochemical screening. Sensitivity analysis to look for influential studies. No study being removed seems to change the heterogeneity or pooled effect – all values are tight to the mid-line for the total pooled estimate, and the CIs overlap.

Figure 5: sensitivity analysis forest plot, post NIPT

Funnel plot for publication bias – there is some asymmetry present, but again there are insufficient numbers to conclude significant publication bias is present.


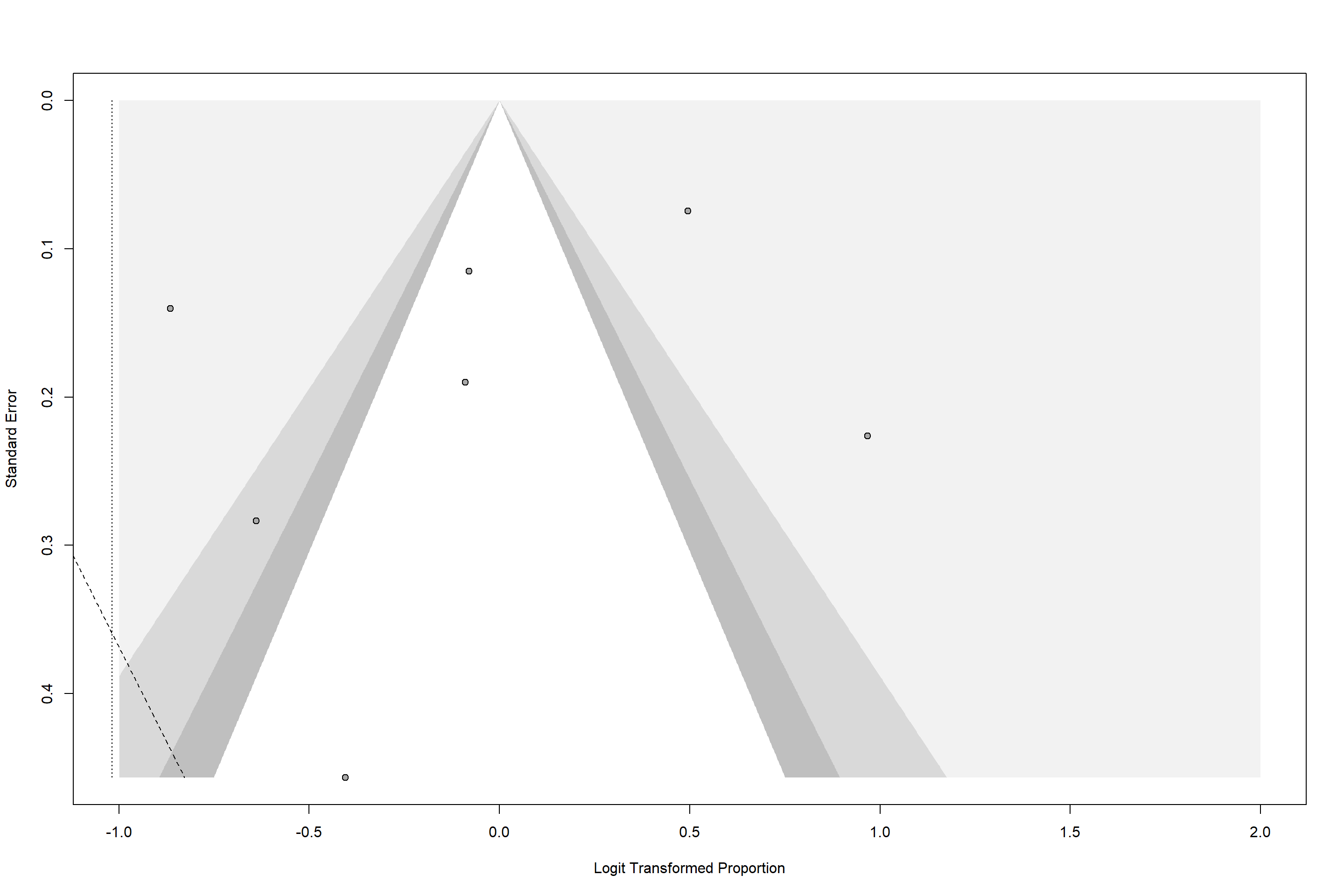


Figure 6: Funnel plot

1.
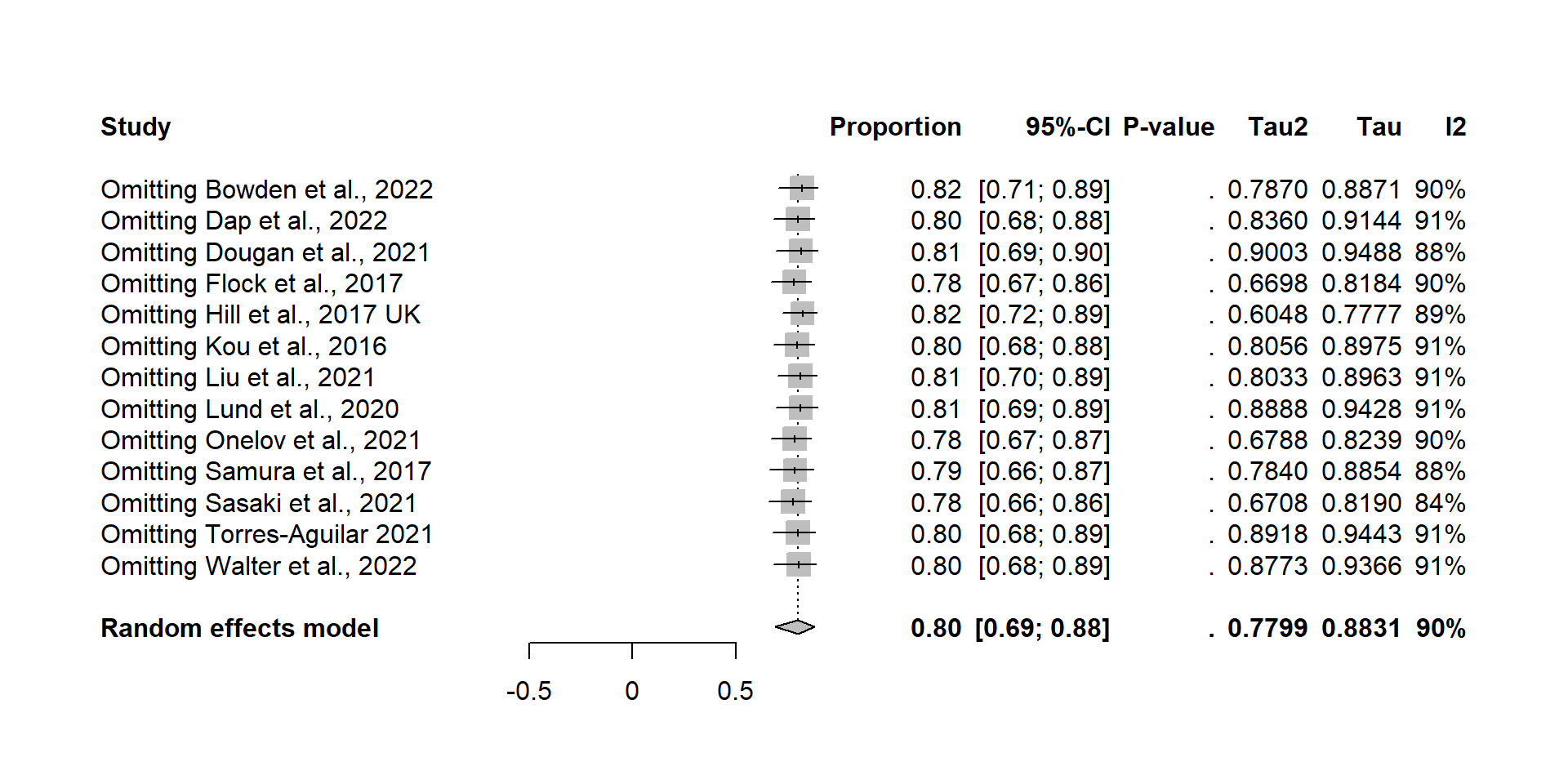
Post-NIPT after 2^nd^ line NIPT high chance

Proportion of high chance pregnancies for DS after NIPT, when offered as a second line screening test, that go on to have an IPD.

Removing Sasaki et al., reduces the heterogeneity to 84%, from 90%, however all pooled estimates are tight around the estimated total and all CIs overlap.

No significant evidence for the removal of Sasaki from the pooled estimate. May be that a small sample size of this study accounts for the reduced heterogeneity when removed.

Figure 7: Sensitivity analysis forest plot, following high chance NIPT result (2^nd^ line screening).

1. Post-NIPT after 1^st^ line NIPT high chance

When NIPT is offered as a first line screening test, proportion who go on to have an IPD after testing high chance.


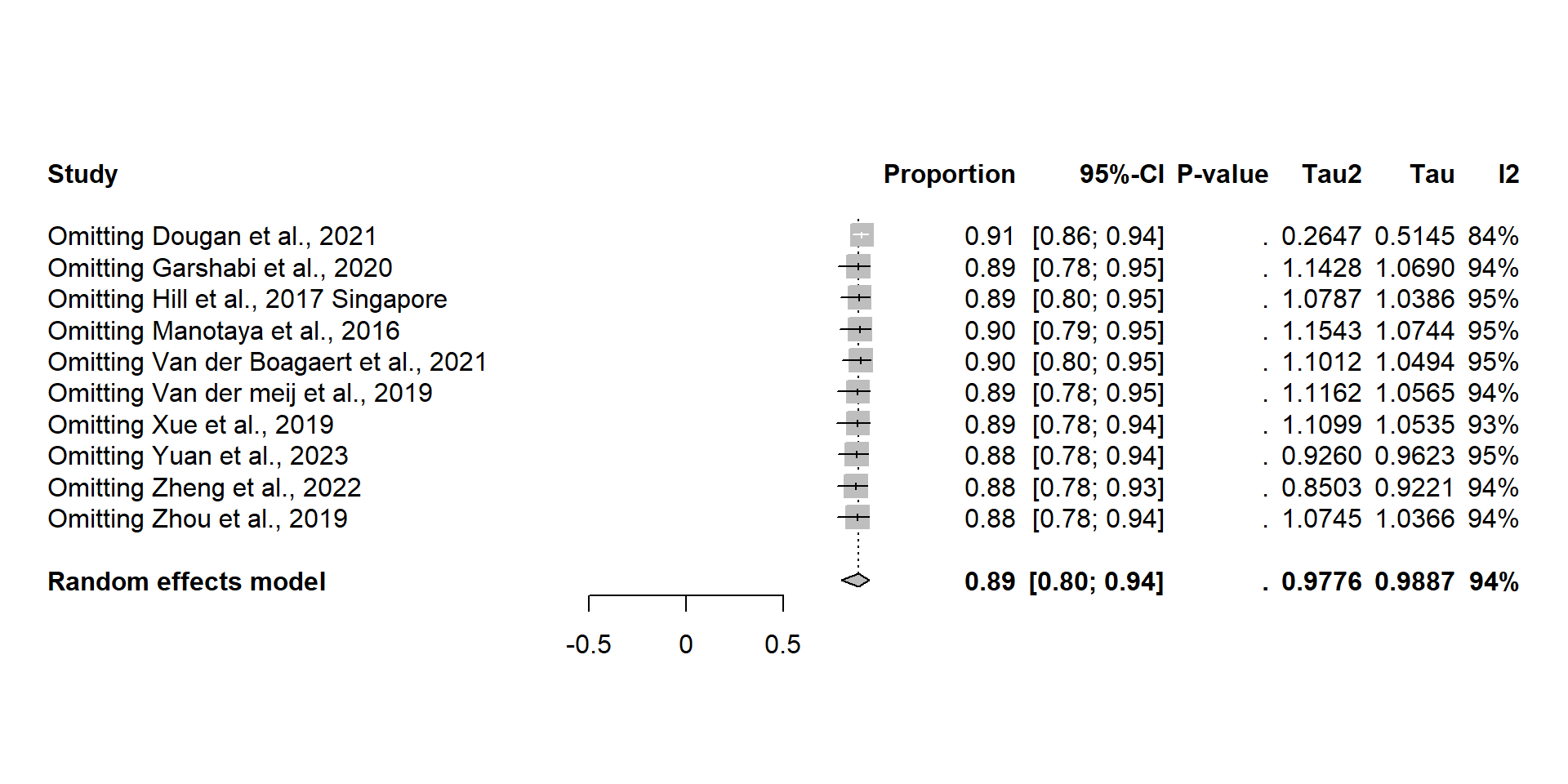


Removing Dougan et al. from model reduced the heterogeneity to 84%, from 94%. There does not seem to be any large effect on the pooled estimate however, and all the CIs overlap. Dougan et al., is a larger study and therefore may have more influence on the heterogeneity but does not seem to influence the overall pooled effect.

Figure 8: Sensitivity analysis forest plot, after high chance NIPT screening (first line)


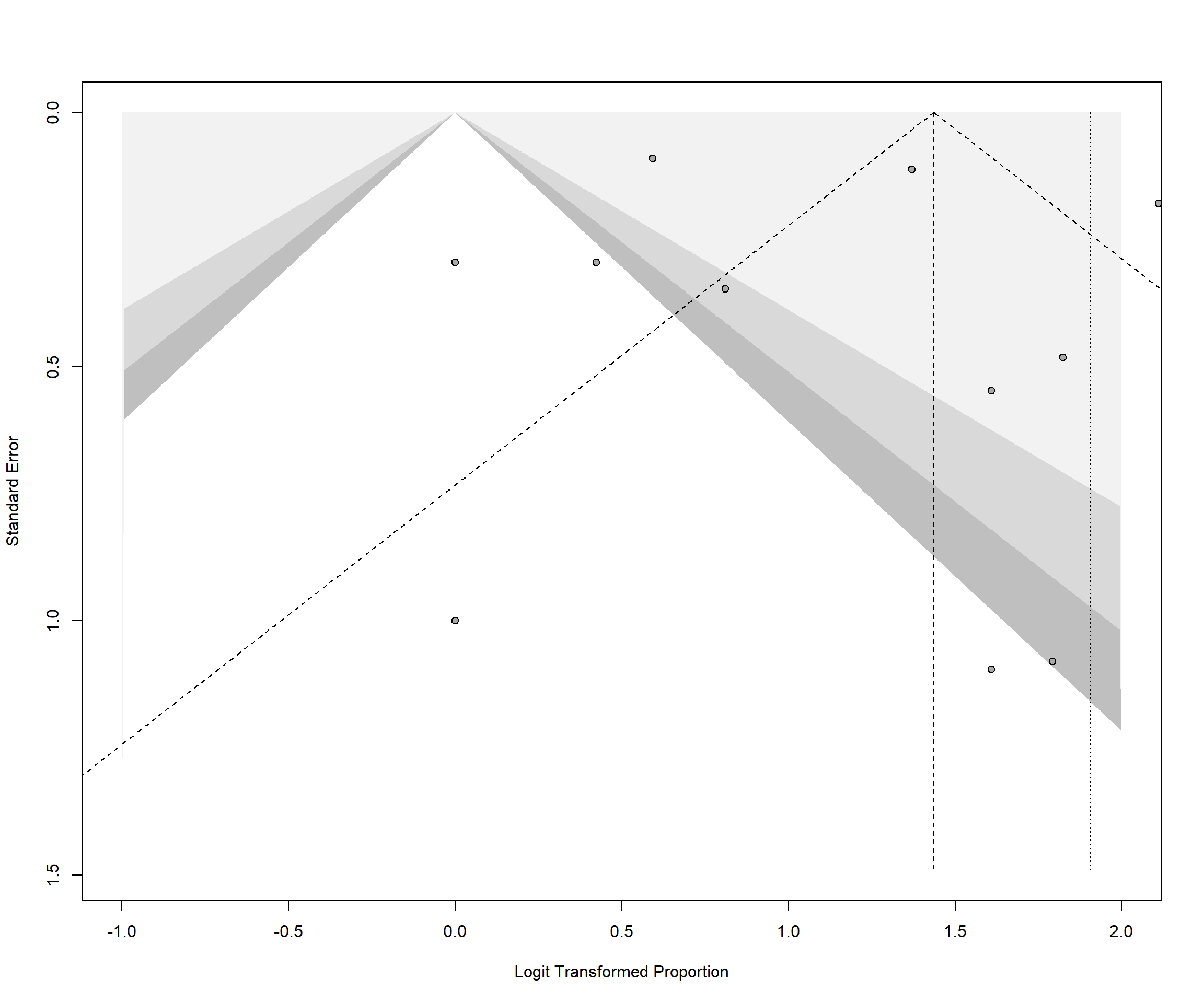
Funnel plot for 1^st^ and 2^nd^ line NIPT high chance going on to have an IPD combined:

Figure 9: Funnel plot

There is lots of asymmetry seen in this plot, this could point to publication bias present in our data. We also need to be aware that asymmetry can be caused by heterogeneity in our data, and we know that our data is extremely heterogenous. Therefore, bias is not the only explanation for this particular presentation of data in the funnel plot.

1. Terminations following high chance NIPT test

Proportion of terminations following high chance NIPT test (first or second line NIPT).


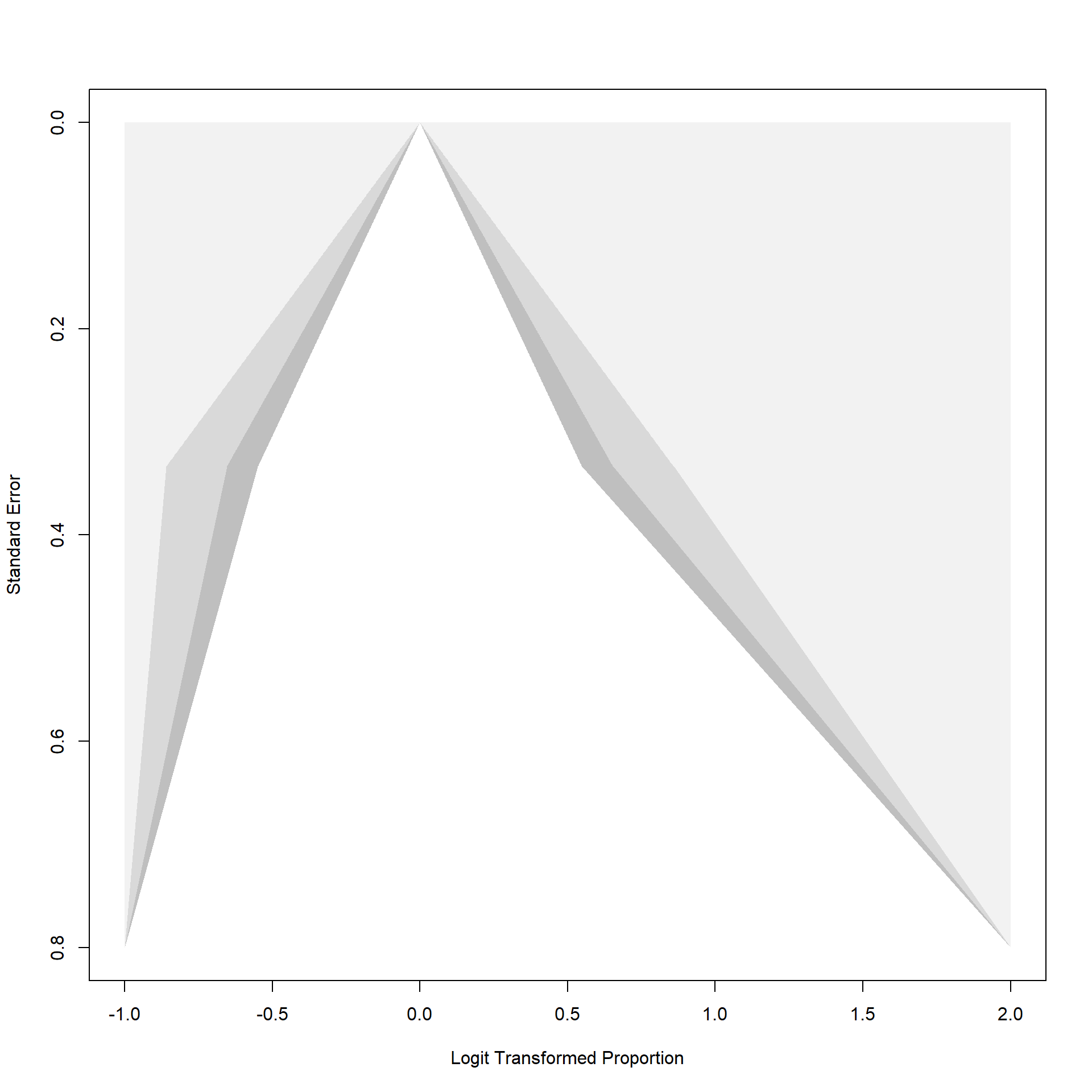

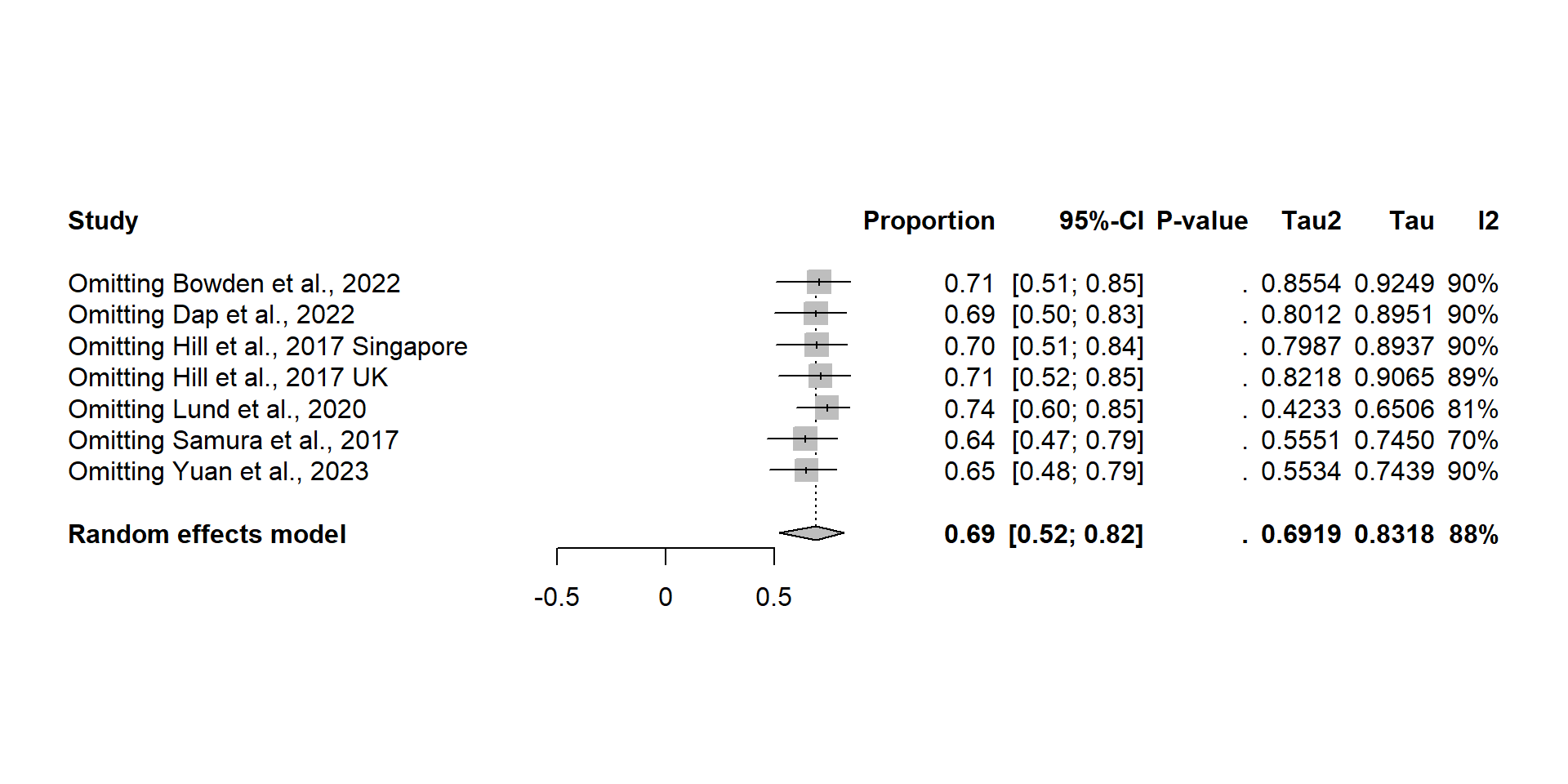
Sensitivity analysis shows removing Samura et al., reduced the heterogeneity to 70% from 88%. This may point to unwanted heterogeneity in the data reported by Samura (small sample size). However, when looking at the pooled effect, there doesn’t seem to be a large effect of removing this study, and all CIs overlap. No findings for the funnel plot of this meta-analysis.

Figure 10: Sensitivity analysis, terminations following high chance NIPT screening

Figure 11: funnel plot

1. Proportion of live births of babies with DS, among women who had a high chance screening result from NIPT.


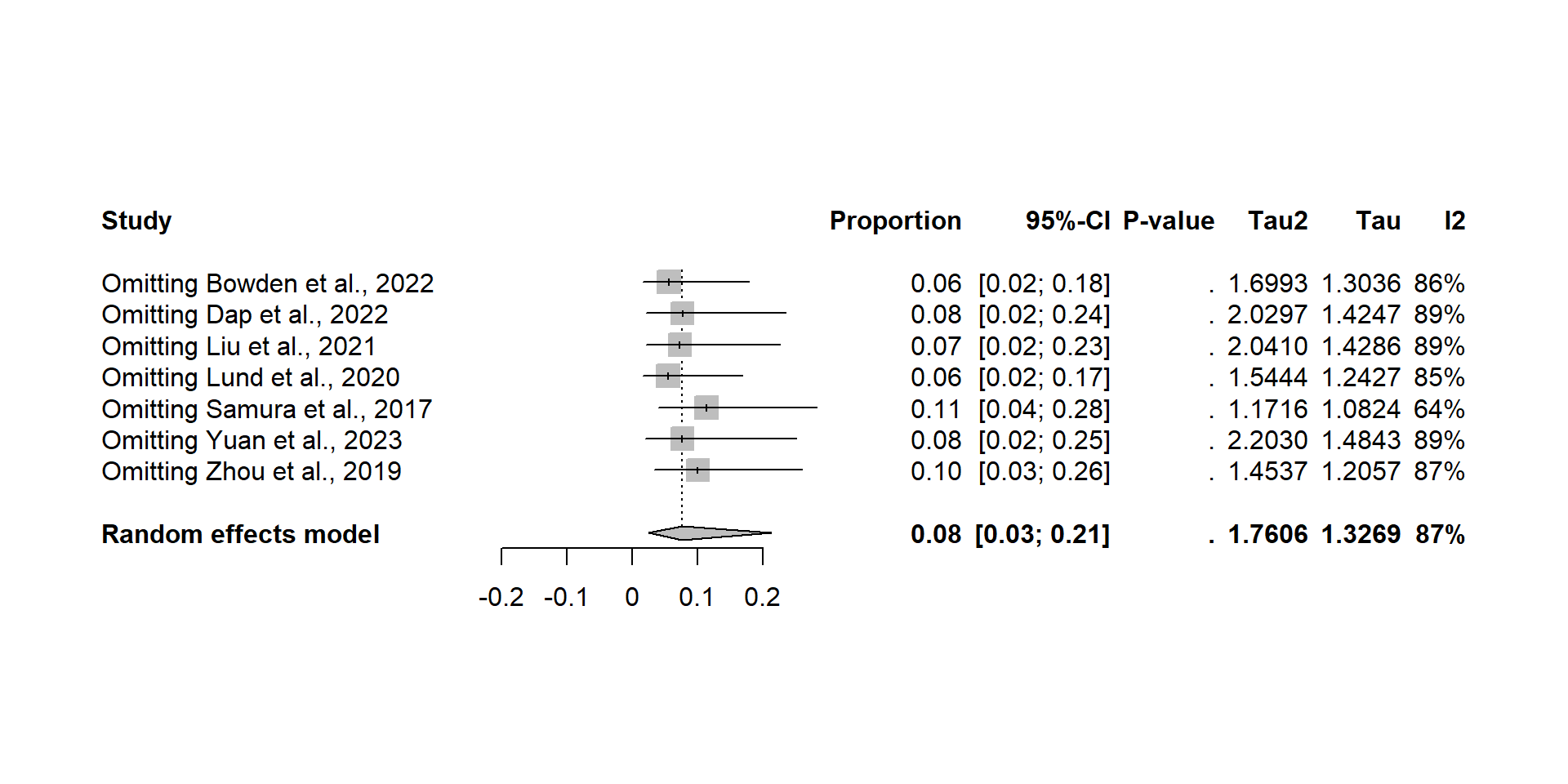
Removing Samura et al again reduces the heterogeneity to 64% - it has a wide CI pointing to small sample size. The removal of this study does not seem to influence the pooled effect significantly (CIs overlap) but the pooled effect is slightly higher without this study included.

Figure 12: sensitivity analysis forest plot, live births following high chance NIPT result.

Asymmetry is seen in the funnel plot which may point to publication bias, although there is an insufficient number of studies to conclude significant publication bias is present.


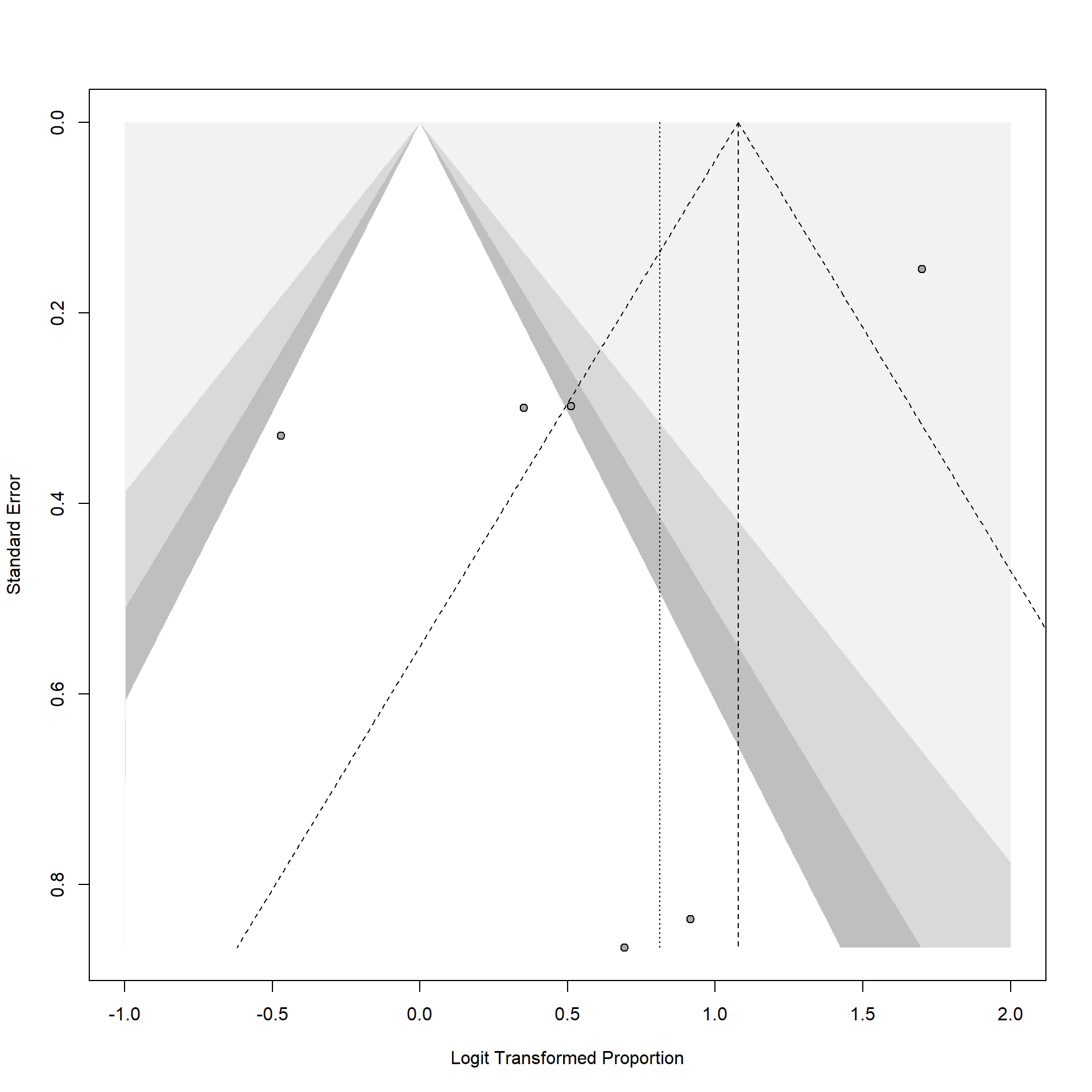


Figure 13: Funnel plot

1. Sensitivity / Subgroup analyses – IPD
2. Subgroup analysis for meta-analysis of proportion of IPDs following higher chance biochemical screening, when NIPT is implemented as a second line screen, adjusting for the risk thresholds used in each study for access to NIPT.

Risk threshold input as a binary variable – studies categorised as having a risk threshold above or below 1:150. When risk threshold is higher than 1:150, the proportion of pregnancies going on to have an IPD is 44.7%. When the risk threshold is lower than 1:150, the number of IPDs is a lower proportion of the high chance population, 19.3%. A lower risk threshold means that women are accessing NIPT when the likelihood they are not actually high chance for DS is higher – these are likely to be picked up by the NIPT test which is more accurate than traditional screening, and therefore a lower number of true positive cases will be in this group. The 95% CI of these two proportions overlap (see below), suggesting there is no significant difference between the groups.

The output below shows that there is no significant difference between groups (p=0.22), despite this difference in proportion.

Results for subgroups (random effects model):

k Proportion 95%-CI tau^2 tau

Risk threshold for NIPT = <1:150 5 0.1932 [0.0584; 0.4805] 2.3621 1.5369

Risk threshold for NIPT = >1:149 3 0.4472 [0.1665; 0.7661] 1.4878 1.2197

Test for subgroup differences (random effects model):

Q d.f. p-value

Between groups 1.50 1 0.2199

1. Subgroup analysis: meta-analysis for number of IPDs following a higher chance NIPT result (1^st^ or 2^nd^ line implementation), adjusting for risk threshold subgroups.

This population is those who have received a high chance result after NIPT screening, accessing it after biochemical screening or as a first line test. ‘None’ = there is no risk threshold as NIPT is implemented as a first line screening test (available to all pregnant women). Above and below 1:150 as above.

The analysis explores if the risk threshold accounts for difference in the proportion of women accessing IPD after a high chance NIPT result for DS. What is reported is that when the threshold is lower than 1:150 (for NIPT as a second line test), 64.9% (95% CI 61%, 68%) of pregnancies opt for IPD, while 94% (95% CI 60.3%, 99.5%) opt for IPD when the risk threshold for NIPT is higher – there is likely to be more positive results in this group. When there is no risk threshold, i.e. NIPT is a first line screen, then 89% (95% CI 86%, 92.7%) opt for IPD. This analysis shows there does seem to be a significant difference in the groups (p<0.0001) and this seems to be between those opting for IPD after a high chance NIPT second line screening, compared to those having IPD from NIPT that was offered as a first line test.

Does the risk threshold for NIPT used affect the number of IPDs women choose after a higher chance first line screening test for DS?

- 1. Higher chance after first line traditional screening (NIPT as second line screen)

Studies were categorised into having a risk threshold that was a risk higher than 1:150, or a threshold lower than 1:150 for access to NIPT. n=5 risk lower than 1:150, n= 3 risk higher than 1:150. Test for difference between subgroups random effect, proportion higher risk group 44.7% went on to IPD, lower risk group 19.3% went on to have IPD. fewer women went on to have IPD if the threshold for NIPT access was lower (letting through women who were not that high chance which was then confirmed by NIPT). There is no significant difference in the groups, as their CIs overlap, p value greater than 0.05.

- 1. After higher chance NIPT screen (first or second line), the access to NIPT would be direct (no threshold) or second line after trad screening. N = 6 studies had threshold risk lower than 1:150, n= 2 had threshold higher than 1:150, n= 9 had no threshold (first line screen). The proportion of women choosing IPD after NIPT higher chance test was 64% for those where the thresholds was set as lower than 1:150, 94% where the threshold was higher than 1:150, and 89% where there was no threshold. There seems to be a significant difference between the subgroups, p <0.0001.

Does when NIPT is offered in the pathway affect the IPD % in women higher chance after NIPT? NIPT either offered as first, second or both. Random effects model, the proportion after first = 90%, second = 81% and first and second = 91%. CI wide, and p value is not significant p = 0.2772.

Meta regression (n=9) for uptake of NIPT for IPD in high-risk DS after traditional screening – r2 = 62% of variability accounted for. Test of moderators p =0.0002, there does seem to be an effect of uptake %. For every 1% increase in uptake, there is a 0.04 decrease (4%) decrease in the proportion of women undertaking IPD.

Results for subgroups (random effects model):

k Proportion 95%-CI tau^2 tau

Risk threshold for NIPT = <1:150 6 0.6493 [0.6116; 0.6853] <0.0001 0.0007

Risk threshold for NIPT = >1:150 2 0.9478 [0.6036; 0.9954] 1.6601 1.2885

Risk threshold for NIPT = None 9 0.8987 [0.8606; 0.9272] 0.1837 0.4286

Test for subgroup differences (random effects model):

Q d.f. p-value

Between groups 62.47 2 < 0.0001

1. Adjusted mean proportion of IPD in the post-NIPT era.

Adjusting for uptake of NIPT by looking at the IPD proportion following biochemical screening at average NIPT uptake. Does uptake % in the population affect the number of IPDs?

Results:

Predicted pooled effect at an average NIPT uptake of 69%, is 32% (95%CI 18%, 57%), this is slightly higher than the 27% undergoing IPD in the pooled proportion of all the data. 95% CI overlap, so this is not significantly different.

1. Subgroup meta-analysis (TOP and live births). Subgroup of studies that report both outcomes in the post NIPT period.

Terminations of pregnancy:


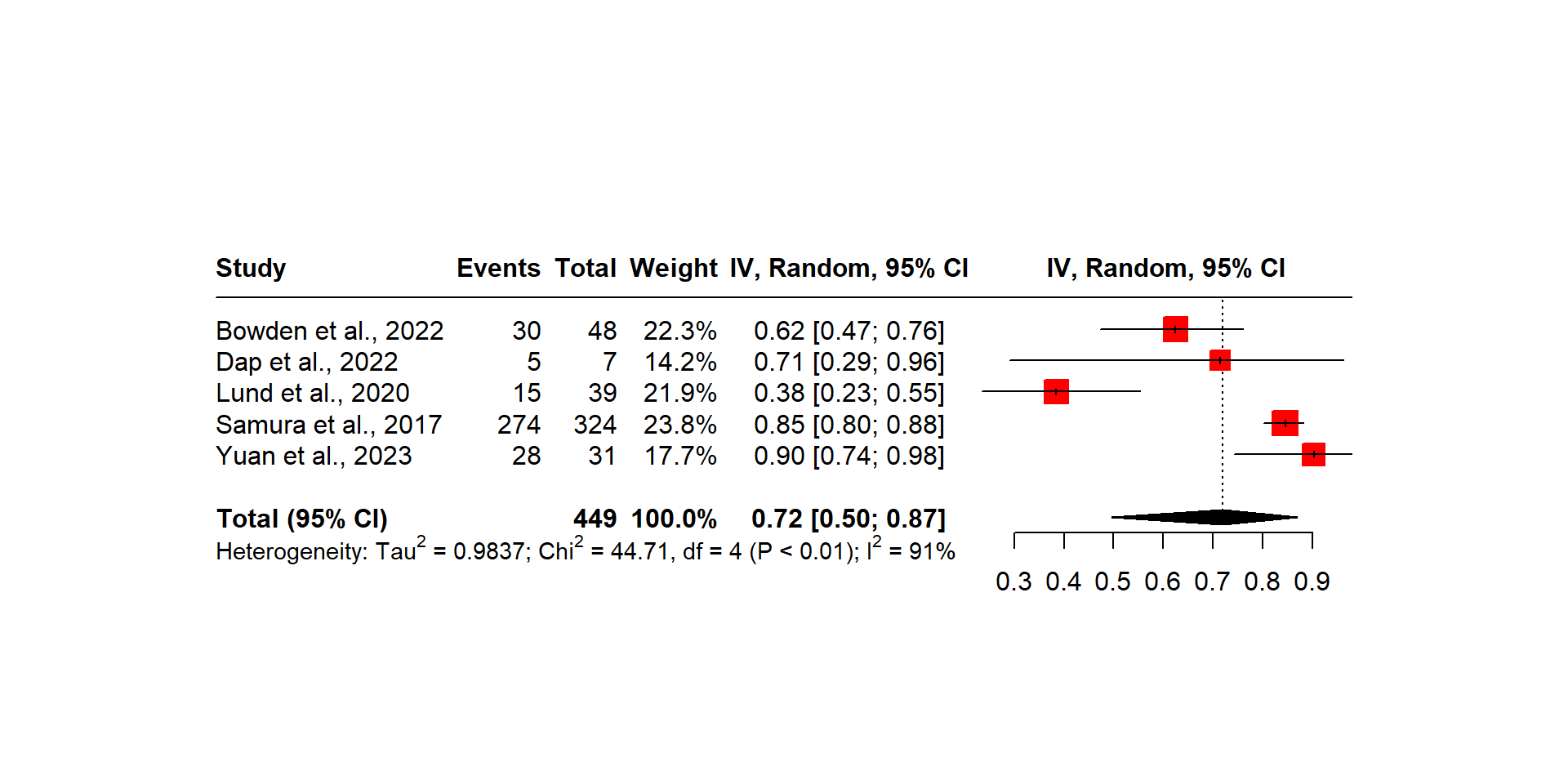


Figure 14: subgroup meta-analysis for termination of pregnancy, for those studies reporting both TOP and live births

Live births:


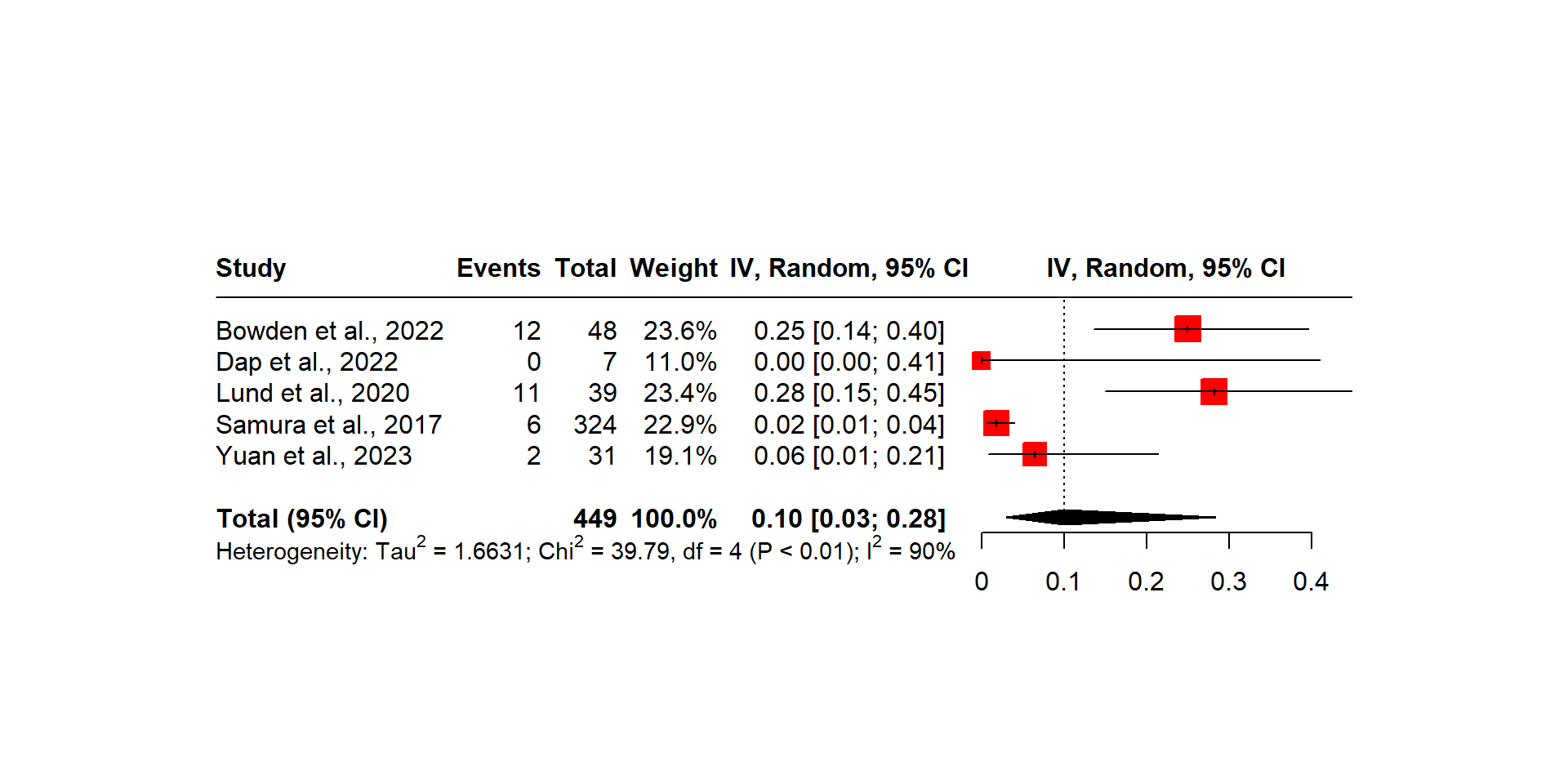


Figure 15: subgroup meta-analysis for live births, for those studies reporting both TOP and live births

1. Risk ratio and Absolute risk reduction

- Risk ratio was calculated, as well as odds ratio, for the difference in IPD between the pre and post NIPT periods:


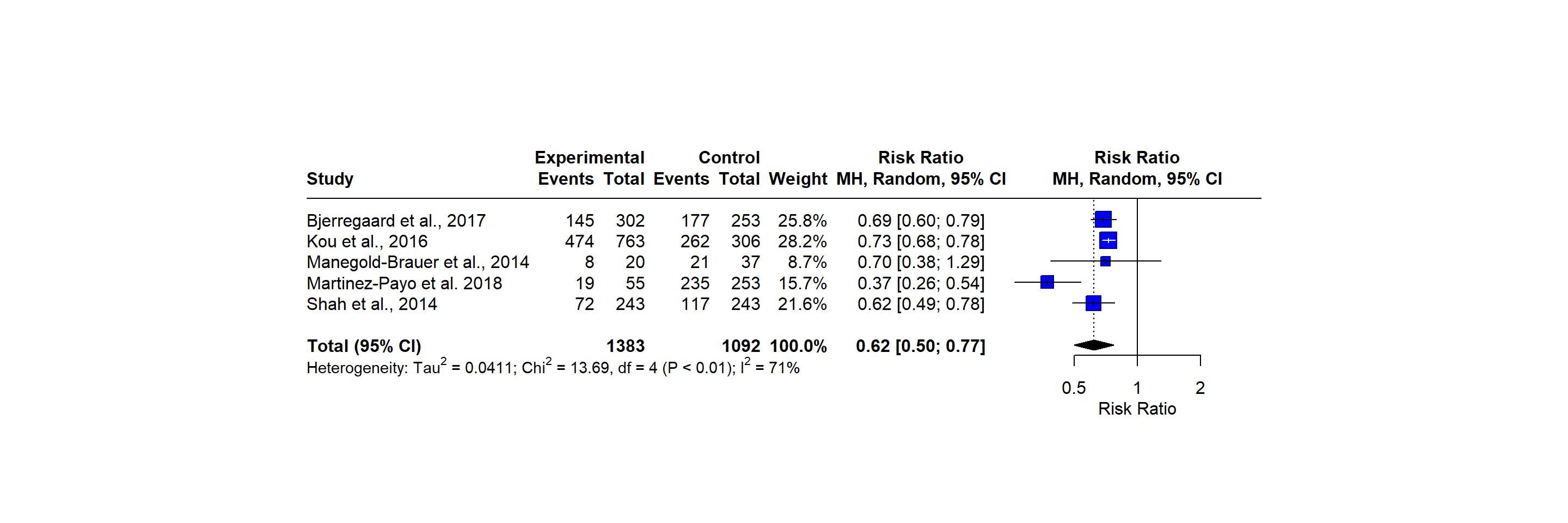


Figure 16: Risk ratio meta-analysis for IPD % pre and post NIPT implementation

- The absolute risk reduction was then calculated from this [100x(1-RR)]:

= ARR of 38%

- Risk difference (between the raw pooled proportion values of either period) 75% - 43% = 32%
