# Supplementary material for: The implementation and impact of non-invasive prenatal testing (NIPT) for Down’s syndrome into antenatal screening programmes: A systematic review and meta-analysis
Source: PLoS One. 2024 May 16;19(5):e0298643. doi: 10.1371/journal.pone.0298643 (PMC11098470; doi:10.1371/journal.pone.0298643)
Supplement: S2 File — Completed PRISMA checklist for systematic review reporting, describing where each point is executed in the systematic review. (DOCX) [file pone.0298643.s005.docx]

| **Section and Topic** | **Item #** | **Checklist item** | **Location where item is reported (page)** |
| --- | --- | --- | --- |
| **TITLE** | | |  |
| Title | 1 | Identify the report as a systematic review. | 1 – ‘The implementation and impact of non-invasive prenatal testing (NIPT) for Down’s syndrome into antenatal screening programmes: a systematic review and meta-analysis’ |
| **ABSTRACT** | | |  |
| Abstract | 2 | See the PRISMA 2020 for Abstracts checklist. | 1 - 2 |
| **INTRODUCTION** | | |  |
| Rationale | 3 | Describe the rationale for the review in the context of existing knowledge. | 5 ‘With new countries implementing NIPT into their national screening programmes every year (8,13,17,18), continual monitoring is important for an up-to-date understanding of the extent of NIPT use in antenatal screening programmes for DS, along with its impact on test uptake and pregnancy outcomes. Therefore, this systematic review will focus on two aspects of NIPT introduction into screening pathways for DS…’ |
| Objectives | 4 | Provide an explicit statement of the objective(s) or question(s) the review addresses. | 5 – under methods, review questions provided as sub-questions for each part of the review. |
| **METHODS** | | |  |
| Eligibility criteria | 5 | Specify the inclusion and exclusion criteria for the review and how studies were grouped for the syntheses. | 6 – ‘We included observational studies and healthcare or governmental publications, from autonomous regions (countries/states/region)…’ criteria given for parts A/B and explanation of grouping for extraction. |
| Information sources | 6 | Specify all databases, registers, websites, organisations, reference lists and other sources searched or consulted to identify studies. Specify the date when each source was last searched or consulted. | 6-7 – ‘Searches were conducted in MEDLINE, Embase, CINAHL and Scopus from January 2010 to March 2023, as the first reported use of NIPT for prenatal testing was in 2011 (1)...’ |
| Search strategy | 7 | Present the full search strategies for all databases, registers and websites, including any filters and limits used. | Supplementary information (S1) |
| Selection process | 8 | Specify the methods used to decide whether a study met the inclusion criteria of the review, including how many reviewers screened each record and each report retrieved, whether they worked independently, and if applicable, details of automation tools used in the process. | 7 – ‘Screening, data extraction, and quality assessment of at least 20% of the papers were carried out in duplicate by independent reviewers…’ |
| Data collection process | 9 | Specify the methods used to collect data from reports, including how many reviewers collected data from each report, whether they worked independently, any processes for obtaining or confirming data from study investigators, and if applicable, details of automation tools used in the process. | 7 – ‘Extracted data was entered into an individual Excel spreadsheet file by each independent reviewer and then merged into a central file once any discrepancies were agreed.’ |
| Data items | 10a | List and define all outcomes for which data were sought. Specify whether all results that were compatible with each outcome domain in each study were sought (e.g. for all measures, time points, analyses), and if not, the methods used to decide which results to collect. | 8 – table 1 provides all outcomes extracted for the two parts of the review. |
|  | 10b | List and define all other variables for which data were sought (e.g. participant and intervention characteristics, funding sources). Describe any assumptions made about any missing or unclear information. | 7- Study characteristics extracted are described here. Authors contacted if missing data required. |
| Study risk of bias assessment | 11 | Specify the methods used to assess risk of bias in the included studies, including details of the tool(s) used, how many reviewers assessed each study and whether they worked independently, and if applicable, details of automation tools used in the process. | 7 – quality assessment / risk of bias with Down’s and Black tool. |
| Effect measures | 12 | Specify for each outcome the effect measure(s) (e.g. risk ratio, mean difference) used in the synthesis or presentation of results. | 8 -9 data synthesis and meta-analysis process. |
| Synthesis methods | 13a | Describe the processes used to decide which studies were eligible for each synthesis (e.g. tabulating the study intervention characteristics and comparing against the planned groups for each synthesis (item #5)). | 9 – ‘Der Simonian and Laird random effects meta-analyses (22) were used to calculate pooled odds ratios (OR) and pooled proportions with respective 95% CI, when there were at least three studies reporting an outcome in part B. |
|  | 13b | Describe any methods required to prepare the data for presentation or synthesis, such as handling of missing summary statistics, or data conversions. | 8-9- ‘Where there was evidence of moderate heterogeneity (I2 > 40%), subgroup analysis’ |
|  | 13c | Describe any methods used to tabulate or visually display results of individual studies and syntheses. | 8 – ‘The data extracted for part A was summarised using…’ |
|  | 13d | Describe any methods used to synthesize results and provide a rationale for the choice(s). If meta-analysis was performed, describe the model(s), method(s) to identify the presence and extent of statistical heterogeneity, and software package(s) used. | 8 - 9 – meta-analysis and regression methods described, heterogeneity and publication bias methods, R used. |
|  | 13e | Describe any methods used to explore possible causes of heterogeneity among study results (e.g. subgroup analysis, meta-regression). | 9 – subgroup analysis method, meta-regression outlined. |
|  | 13f | Describe any sensitivity analyses conducted to assess robustness of the synthesized results. | 9 – ‘Sensitivity analysis was performed for each of the meta-analyses to identify the effect of individual studies on the pooled effect size and between-study heterogeneity. Meta-analysis results were visualised using forest plots. All analyses were conducted in R v4.1.3.’ |
| Reporting bias assessment | 14 | Describe any methods used to assess risk of bias due to missing results in a synthesis (arising from reporting biases). | None described |
| Certainty assessment | 15 | Describe any methods used to assess certainty (or confidence) in the body of evidence for an outcome. | 8 – ‘95% CI reported’ |
| **RESULTS** | | |  |
| Study selection | 16a | Describe the results of the search and selection process, from the number of records identified in the search to the number of studies included in the review, ideally using a flow diagram. | 9 – 10 – flow diagram and description of included studies |
|  | 16b | Cite studies that might appear to meet the inclusion criteria, but which were excluded, and explain why they were excluded. | 10 – flow diagram gives reasons for exclusion at full text stage |
| Study characteristics | 17 | Cite each included study and present its characteristics. | S1 appendix – characteristics of included studies |
| Risk of bias in studies | 18 | Present assessments of risk of bias for each included study. | S1 appendix – risk of bias presented for each paper in table 1. |
| Results of individual studies | 19 | For all outcomes, present, for each study: (a) summary statistics for each group (where appropriate) and (b) an effect estimate and its precision (e.g. confidence/credible interval), ideally using structured tables or plots. | 17 – uptake of NIPT results, and part B outcomes given as summary statistics and meta-analysis results. |
| Results of syntheses | 20a | For each synthesis, briefly summarise the characteristics and risk of bias among contributing studies. | 12 + 19 – parts A and B initial results, describing characteristics of the papers |
|  | 20b | Present results of all statistical syntheses conducted. If meta-analysis was done, present for each the summary estimate and its precision (e.g. confidence/credible interval) and measures of statistical heterogeneity. If comparing groups, describe the direction of the effect. | 18 - 21 - Part B |
|  | 20c | Present results of all investigations of possible causes of heterogeneity among study results. | 18-25 - Presented for part B following each meta-analysis |
|  | 20d | Present results of all sensitivity analyses conducted to assess the robustness of the synthesized results. | S3 appendix - part B data analysis |
| Reporting biases | 21 | Present assessments of risk of bias due to missing results (arising from reporting biases) for each synthesis assessed. | S3 appendix – funnel plots described |
| Certainty of evidence | 22 | Present assessments of certainty (or confidence) in the body of evidence for each outcome assessed. | P values and 95% CI presented for each analyses (results section) |
| **DISCUSSION** | | |  |
| Discussion | 23a | Provide a general interpretation of the results in the context of other evidence. | 27 – ‘comparison with existing literature’ |
|  | 23b | Discuss any limitations of the evidence included in the review. | 26 – ‘limitations’ |
|  | 23c | Discuss any limitations of the review processes used. | 26 – ‘Limitations’ |
|  | 23d | Discuss implications of the results for practice, policy, and future research. | 28 – ‘implications for clinical practise / policy’ |
| **OTHER INFORMATION** | | |  |
| Registration and protocol | 24a | Provide registration information for the review, including register name and registration number, or state that the review was not registered. | 7 – ‘Full details of the search strategy can be found as part of the PROSPERO registered protocol (CRD42022306167). ‘ |
|  | 24b | Indicate where the review protocol can be accessed, or state that a protocol was not prepared. | 7 – see above |
|  | 24c | Describe and explain any amendments to information provided at registration or in the protocol. | Amendments made in the PROSPERO registered protocol |
| Support | 25 | Describe sources of financial or non-financial support for the review, and the role of the funders or sponsors in the review. | 42 |
| Competing interests | 26 | Declare any competing interests of review authors. | 42 |
| Availability of data, code and other materials | 27 | Report which of the following are publicly available and where they can be found: template data collection forms; data extracted from included studies; data used for all analyses; analytic code; any other materials used in the review. | Data used for all analyses can be requested from corresponding author |

*From:*  Page MJ, McKenzie JE, Bossuyt PM, Boutron I, Hoffmann TC, Mulrow CD, et al. The PRISMA 2020 statement: an updated guideline for reporting systematic reviews. BMJ 2021;372:n71. doi: 10.1136/bmj.n71

For more information, visit: <http://www.prisma-statement.org/>
