# Supplementary material for: The implementation and impact of non-invasive prenatal testing (NIPT) for Down’s syndrome into antenatal screening programmes: A systematic review and meta-analysis
Source: PLoS One. 2024 May 16;19(5):e0298643. doi: 10.1371/journal.pone.0298643 (PMC11098470; doi:10.1371/journal.pone.0298643)
Supplement: Attachment — Submitted filename: Response to Reviewers.docx [file pone.0298643.s007.docx]

Dear Editor,

Thank you for the opportunity to revise and resubmit our manuscript ‘The implementation and impact of non-invasive prenatal testing (NIPT) for Down’s syndrome into antenatal screening programmes: a systematic review and meta-analysis’ for publication in *PLOS ONE*. We appreciate the time dedicated by you and the reviewers to provide feedback on the manuscript, offering insightful comments and valuable improvements to make. We have revised the manuscript to reflect the reviewers’ comments, which are sign-posted in the track changes version of the manuscript attached. We have also provided a point-by-point response to the reviewers’ comments below.

1. **Academic editor**

Journal Requirements:

1. When submitting your revision, we need you to address these additional requirements.

Please ensure that your manuscript meets PLOS ONE's style requirements, including those for file naming. The PLOS ONE style templates can be found at 
[https://journals.plos.org/plosone/s/file?id=wjVg/PLOSOne_formatting_sample_main_body.pdf](https://eur03.safelinks.protection.outlook.com/?url=https%3A%2F%2Fjournals.plos.org%2Fplosone%2Fs%2Ffile%3Fid%3DwjVg%2FPLOSOne_formatting_sample_main_body.pdf&data=05%7C01%7Ce.sebire.21%40abdn.ac.uk%7Caf6ba56de2fd45b8d16b08dbeb4fd3d7%7C8c2b19ad5f9c49d490773ec3cfc52b3f%7C0%7C0%7C638362500315666484%7CUnknown%7CTWFpbGZsb3d8eyJWIjoiMC4wLjAwMDAiLCJQIjoiV2luMzIiLCJBTiI6Ik1haWwiLCJXVCI6Mn0%3D%7C3000%7C%7C%7C&sdata=C4ivk9hq4qD4gzoLkRdyn40y5gZQzeocEAREv0CgINc%3D&reserved=0) and 
[https://journals.plos.org/plosone/s/file?id=ba62/PLOSOne_formatting_sample_title_authors_affiliations.pdf](https://eur03.safelinks.protection.outlook.com/?url=https%3A%2F%2Fjournals.plos.org%2Fplosone%2Fs%2Ffile%3Fid%3Dba62%2FPLOSOne_formatting_sample_title_authors_affiliations.pdf&data=05%7C01%7Ce.sebire.21%40abdn.ac.uk%7Caf6ba56de2fd45b8d16b08dbeb4fd3d7%7C8c2b19ad5f9c49d490773ec3cfc52b3f%7C0%7C0%7C638362500315822736%7CUnknown%7CTWFpbGZsb3d8eyJWIjoiMC4wLjAwMDAiLCJQIjoiV2luMzIiLCJBTiI6Ik1haWwiLCJXVCI6Mn0%3D%7C3000%7C%7C%7C&sdata=BLfbcDtOBfEqZIo%2FnAgGcZS8XjbYkQd2E8z9XjDfo%2FA%3D&reserved=0)

Thank you for highlighting this, we have actioned the style requirements according to the templates you have given.

2. Please ensure that your PRISMA flow diagram is included in your main manuscript file as Figure 1; please see the PLOS ONE submission guidelines for systematic reviews and meta-analyses at [https://journals.plos.org/plosone/s/submission-guidelines#loc-systematic-reviews-and-meta-analyses](https://eur03.safelinks.protection.outlook.com/?url=https%3A%2F%2Fjournals.plos.org%2Fplosone%2Fs%2Fsubmission-guidelines%23loc-systematic-reviews-and-meta-analyses&data=05%7C01%7Ce.sebire.21%40abdn.ac.uk%7Caf6ba56de2fd45b8d16b08dbeb4fd3d7%7C8c2b19ad5f9c49d490773ec3cfc52b3f%7C0%7C0%7C638362500315822736%7CUnknown%7CTWFpbGZsb3d8eyJWIjoiMC4wLjAwMDAiLCJQIjoiV2luMzIiLCJBTiI6Ik1haWwiLCJXVCI6Mn0%3D%7C3000%7C%7C%7C&sdata=tq20L7LVAwbk1x%2F1kQvywdUMDW9HtrEUG7gs1Ir3zgQ%3D&reserved=0).

Thank you for this comment. We had initially included the PRISMA flow diagram as Figure 2 as we used a figure in the introduction to help explain the screening pathways. We do feel this introductory figure is important, as many of the readership may not be familiar with the screening pathways being described and wonder if the journal editors have any suggestions on how it can still be incorporated in the Introduction. For this draft we have removed the original Figure 1 so that the PRISMA diagram is now Figure 1. We have placed the screening pathway figure as Supplementary Figure 1.

3. In your Data Availability statement, you have not specified where the minimal data set underlying the results described in your manuscript can be found. PLOS defines a study's minimal data set as the underlying data used to reach the conclusions drawn in the manuscript and any additional data required to replicate the reported study findings in their entirety. All PLOS journals require that the minimal data set be made fully available. For more information about our data policy, please see [http://journals.plos.org/plosone/s/data-availability](https://eur03.safelinks.protection.outlook.com/?url=http%3A%2F%2Fjournals.plos.org%2Fplosone%2Fs%2Fdata-availability&data=05%7C01%7Ce.sebire.21%40abdn.ac.uk%7Caf6ba56de2fd45b8d16b08dbeb4fd3d7%7C8c2b19ad5f9c49d490773ec3cfc52b3f%7C0%7C0%7C638362500315822736%7CUnknown%7CTWFpbGZsb3d8eyJWIjoiMC4wLjAwMDAiLCJQIjoiV2luMzIiLCJBTiI6Ik1haWwiLCJXVCI6Mn0%3D%7C3000%7C%7C%7C&sdata=zMXnahIM1Z7MeQJ0rzs%2BR4HzrV2dkWNrZMhlgR3j5k8%3D&reserved=0).

Upon re-submitting your revised manuscript, please upload your study’s minimal underlying data set as either Supporting Information files or to a stable, public repository and include the relevant URLs, DOIs, or accession numbers within your revised cover letter. For a list of acceptable repositories, please see [http://journals.plos.org/plosone/s/data-availability#loc-recommended-repositories](https://eur03.safelinks.protection.outlook.com/?url=http%3A%2F%2Fjournals.plos.org%2Fplosone%2Fs%2Fdata-availability%23loc-recommended-repositories&data=05%7C01%7Ce.sebire.21%40abdn.ac.uk%7Caf6ba56de2fd45b8d16b08dbeb4fd3d7%7C8c2b19ad5f9c49d490773ec3cfc52b3f%7C0%7C0%7C638362500315822736%7CUnknown%7CTWFpbGZsb3d8eyJWIjoiMC4wLjAwMDAiLCJQIjoiV2luMzIiLCJBTiI6Ik1haWwiLCJXVCI6Mn0%3D%7C3000%7C%7C%7C&sdata=%2BMCYguqSX%2FgylmzxHmA4ZsmiWdqGs2eR0P24XumGGmE%3D&reserved=0). Any potentially identifying patient information must be fully anonymized.

Important: If there are ethical or legal restrictions to sharing your data publicly, please explain these restrictions in detail. Please see our guidelines for more information on what we consider unacceptable restrictions to publicly sharing data: [http://journals.plos.org/plosone/s/data-availability#loc-unacceptable-data-access-restrictions](https://eur03.safelinks.protection.outlook.com/?url=http%3A%2F%2Fjournals.plos.org%2Fplosone%2Fs%2Fdata-availability%23loc-unacceptable-data-access-restrictions&data=05%7C01%7Ce.sebire.21%40abdn.ac.uk%7Caf6ba56de2fd45b8d16b08dbeb4fd3d7%7C8c2b19ad5f9c49d490773ec3cfc52b3f%7C0%7C0%7C638362500315822736%7CUnknown%7CTWFpbGZsb3d8eyJWIjoiMC4wLjAwMDAiLCJQIjoiV2luMzIiLCJBTiI6Ik1haWwiLCJXVCI6Mn0%3D%7C3000%7C%7C%7C&sdata=54%2FiVU5POYkOGasR9oDxcnskIEtwDd9%2BJHU2437SNtI%3D&reserved=0). Note that it is not acceptable for the authors to be the sole named individuals responsible for ensuring data access.

We will update your Data Availability statement to reflect the information you provide in your cover letter.

Thank you for highlighting this. For part A of the review, all data is available in Tables 3 and 4. The data used for the meta-analyses in part B is incorporated in the forest plots or in supplementary file 3. We have also now made the whole dataset for the underlying results available in a project directory held in Open Science Framework repository.

The URL for where this dataset is held is: <https://osf.io/85qg4/?view_only=179cbc7b4acc4a1cbbcf19d6b8dca3b5>

4. We note that you have stated that you will provide repository information for your data at acceptance. Should your manuscript be accepted for publication, we will hold it until you provide the relevant accession numbers or DOIs necessary to access your data. If you wish to make changes to your Data Availability statement, please describe these changes in your cover letter and we will update your Data Availability statement to reflect the information you provide.

Thank you for your comment, we have made the dataset available in a repository in line with our data availability statement, URL: <https://osf.io/85qg4/?view_only=179cbc7b4acc4a1cbbcf19d6b8dca3b5>

5. Please amend either the abstract on the online submission form (via Edit Submission) or the abstract in the manuscript so that they are identical.

Thank you for bringing this to our attention, we have amended the abstracts so that they are identical.

6. We note that Figure 3 in your submission contain map images which may be copyrighted. All PLOS content is published under the Creative Commons Attribution License (CC BY 4.0), which means that the manuscript, images, and Supporting Information files will be freely available online, and any third party is permitted to access, download, copy, distribute, and use these materials in any way, even commercially, with proper attribution. For these reasons, we cannot publish previously copyrighted maps or satellite images created using proprietary data, such as Google software (Google Maps, Street View, and Earth). For more information, see our copyright guidelines: [http://journals.plos.org/plosone/s/licenses-and-copyright](https://eur03.safelinks.protection.outlook.com/?url=http%3A%2F%2Fjournals.plos.org%2Fplosone%2Fs%2Flicenses-and-copyright&data=05%7C01%7Ce.sebire.21%40abdn.ac.uk%7Caf6ba56de2fd45b8d16b08dbeb4fd3d7%7C8c2b19ad5f9c49d490773ec3cfc52b3f%7C0%7C0%7C638362500315822736%7CUnknown%7CTWFpbGZsb3d8eyJWIjoiMC4wLjAwMDAiLCJQIjoiV2luMzIiLCJBTiI6Ik1haWwiLCJXVCI6Mn0%3D%7C3000%7C%7C%7C&sdata=UA3NPuJUbfmN4VNfbKWTIQ1mOICM7LTOLFo4puwvzB8%3D&reserved=0).
 
We require you to either present written permission from the copyright holder to publish these figures specifically under the CC BY 4.0 license, or (2) remove the figures from your submission:
 
a. You may seek permission from the original copyright holder of Figure 3 to publish the content specifically under the CC BY 4.0 license.  
 
We recommend that you contact the original copyright holder with the Content Permission Form ([http://journals.plos.org/plosone/s/file?id=7c09/content-permission-form.pdf](https://eur03.safelinks.protection.outlook.com/?url=http%3A%2F%2Fjournals.plos.org%2Fplosone%2Fs%2Ffile%3Fid%3D7c09%2Fcontent-permission-form.pdf&data=05%7C01%7Ce.sebire.21%40abdn.ac.uk%7Caf6ba56de2fd45b8d16b08dbeb4fd3d7%7C8c2b19ad5f9c49d490773ec3cfc52b3f%7C0%7C0%7C638362500315822736%7CUnknown%7CTWFpbGZsb3d8eyJWIjoiMC4wLjAwMDAiLCJQIjoiV2luMzIiLCJBTiI6Ik1haWwiLCJXVCI6Mn0%3D%7C3000%7C%7C%7C&sdata=rxfJVPxyqjK09ZrYkWXVhiQ4Dha1JncbihRvZxaQspM%3D&reserved=0)) and the following text:
“I request permission for the open-access journal PLOS ONE to publish XXX under the Creative Commons Attribution License (CCAL) CC BY 4.0 ([http://creativecommons.org/licenses/by/4.0/](https://eur03.safelinks.protection.outlook.com/?url=http%3A%2F%2Fcreativecommons.org%2Flicenses%2Fby%2F4.0%2F&data=05%7C01%7Ce.sebire.21%40abdn.ac.uk%7Caf6ba56de2fd45b8d16b08dbeb4fd3d7%7C8c2b19ad5f9c49d490773ec3cfc52b3f%7C0%7C0%7C638362500315978973%7CUnknown%7CTWFpbGZsb3d8eyJWIjoiMC4wLjAwMDAiLCJQIjoiV2luMzIiLCJBTiI6Ik1haWwiLCJXVCI6Mn0%3D%7C3000%7C%7C%7C&sdata=rI8KIANZrNkTQGaWCG%2FVq%2B%2FLXzDuaRb5C5BhuAzS%2FiI%3D&reserved=0)). Please be aware that this license allows unrestricted use and distribution, even commercially, by third parties. Please reply and provide explicit written permission to publish XXX under a CC BY license and complete the attached form.”
 
Please upload the completed Content Permission Form or other proof of granted permissions as an "Other" file with your submission.
 
In the figure caption of the copyrighted figure, please include the following text: “Reprinted from [ref] under a CC BY license, with permission from [name of publisher], original copyright [original copyright year].”
 
b. If you are unable to obtain permission from the original copyright holder to publish these figures under the CC BY 4.0 license or if the copyright holder’s requirements are incompatible with the CC BY 4.0 license, please either i) remove the figure or ii) supply a replacement figure that complies with the CC BY 4.0 license. Please check copyright information on all replacement figures and update the figure caption with source information. If applicable, please specify in the figure caption text when a figure is similar but not identical to the original image and is therefore for illustrative purposes only.
The following resources for replacing copyrighted map figures may be helpful:
 
USGS National Map Viewer (public domain): [http://viewer.nationalmap.gov/viewer/](https://eur03.safelinks.protection.outlook.com/?url=http%3A%2F%2Fviewer.nationalmap.gov%2Fviewer%2F&data=05%7C01%7Ce.sebire.21%40abdn.ac.uk%7Caf6ba56de2fd45b8d16b08dbeb4fd3d7%7C8c2b19ad5f9c49d490773ec3cfc52b3f%7C0%7C0%7C638362500315978973%7CUnknown%7CTWFpbGZsb3d8eyJWIjoiMC4wLjAwMDAiLCJQIjoiV2luMzIiLCJBTiI6Ik1haWwiLCJXVCI6Mn0%3D%7C3000%7C%7C%7C&sdata=SsrnX79jb1KUQ4s%2BMvSWsypHLfWIAf5SkJQmICk8n7A%3D&reserved=0)
The Gateway to Astronaut Photography of Earth (public domain): [http://eol.jsc.nasa.gov/sseop/clickmap/](https://eur03.safelinks.protection.outlook.com/?url=http%3A%2F%2Feol.jsc.nasa.gov%2Fsseop%2Fclickmap%2F&data=05%7C01%7Ce.sebire.21%40abdn.ac.uk%7Caf6ba56de2fd45b8d16b08dbeb4fd3d7%7C8c2b19ad5f9c49d490773ec3cfc52b3f%7C0%7C0%7C638362500315978973%7CUnknown%7CTWFpbGZsb3d8eyJWIjoiMC4wLjAwMDAiLCJQIjoiV2luMzIiLCJBTiI6Ik1haWwiLCJXVCI6Mn0%3D%7C3000%7C%7C%7C&sdata=ajufiqv97xUBO3WyPxhXN%2BnLcywM5DCTYpOdpbj%2FnCI%3D&reserved=0)
Maps at the CIA (public domain): [https://www.cia.gov/library/publications/the-world-factbook/index.html](https://eur03.safelinks.protection.outlook.com/?url=https%3A%2F%2Fwww.cia.gov%2Flibrary%2Fpublications%2Fthe-world-factbook%2Findex.html&data=05%7C01%7Ce.sebire.21%40abdn.ac.uk%7Caf6ba56de2fd45b8d16b08dbeb4fd3d7%7C8c2b19ad5f9c49d490773ec3cfc52b3f%7C0%7C0%7C638362500315978973%7CUnknown%7CTWFpbGZsb3d8eyJWIjoiMC4wLjAwMDAiLCJQIjoiV2luMzIiLCJBTiI6Ik1haWwiLCJXVCI6Mn0%3D%7C3000%7C%7C%7C&sdata=DG4mLMTT37jtC%2Fd1cRx9BYGr7gtQ1vdrZLH4c6PLUSw%3D&reserved=0) and [https://www.cia.gov/library/publications/cia-maps-publications/index.html](https://eur03.safelinks.protection.outlook.com/?url=https%3A%2F%2Fwww.cia.gov%2Flibrary%2Fpublications%2Fcia-maps-publications%2Findex.html&data=05%7C01%7Ce.sebire.21%40abdn.ac.uk%7Caf6ba56de2fd45b8d16b08dbeb4fd3d7%7C8c2b19ad5f9c49d490773ec3cfc52b3f%7C0%7C0%7C638362500315978973%7CUnknown%7CTWFpbGZsb3d8eyJWIjoiMC4wLjAwMDAiLCJQIjoiV2luMzIiLCJBTiI6Ik1haWwiLCJXVCI6Mn0%3D%7C3000%7C%7C%7C&sdata=KecIYg6P9ui8R7abphXTQwgwXVsI8rqCveCX8W2uMHU%3D&reserved=0)
NASA Earth Observatory (public domain): [http://earthobservatory.nasa.gov/](https://eur03.safelinks.protection.outlook.com/?url=http%3A%2F%2Fearthobservatory.nasa.gov%2F&data=05%7C01%7Ce.sebire.21%40abdn.ac.uk%7Caf6ba56de2fd45b8d16b08dbeb4fd3d7%7C8c2b19ad5f9c49d490773ec3cfc52b3f%7C0%7C0%7C638362500315978973%7CUnknown%7CTWFpbGZsb3d8eyJWIjoiMC4wLjAwMDAiLCJQIjoiV2luMzIiLCJBTiI6Ik1haWwiLCJXVCI6Mn0%3D%7C3000%7C%7C%7C&sdata=7Zh8XK2IxFTeZ%2FYdgoMAbX4V5LJsld3weIOMd%2Fqf3CA%3D&reserved=0)
Landsat: [http://landsat.visibleearth.nasa.gov/](https://eur03.safelinks.protection.outlook.com/?url=http%3A%2F%2Flandsat.visibleearth.nasa.gov%2F&data=05%7C01%7Ce.sebire.21%40abdn.ac.uk%7Caf6ba56de2fd45b8d16b08dbeb4fd3d7%7C8c2b19ad5f9c49d490773ec3cfc52b3f%7C0%7C0%7C638362500315978973%7CUnknown%7CTWFpbGZsb3d8eyJWIjoiMC4wLjAwMDAiLCJQIjoiV2luMzIiLCJBTiI6Ik1haWwiLCJXVCI6Mn0%3D%7C3000%7C%7C%7C&sdata=x%2FYyy0OBDlmWKNO17bw2hTSX6TCQB4zprlBVj%2BpzxrQ%3D&reserved=0)
USGS EROS (Earth Resources Observatory and Science (EROS) Center) (public domain): [http://eros.usgs.gov/#](https://eur03.safelinks.protection.outlook.com/?url=http%3A%2F%2Feros.usgs.gov%2F%23&data=05%7C01%7Ce.sebire.21%40abdn.ac.uk%7Caf6ba56de2fd45b8d16b08dbeb4fd3d7%7C8c2b19ad5f9c49d490773ec3cfc52b3f%7C0%7C0%7C638362500315978973%7CUnknown%7CTWFpbGZsb3d8eyJWIjoiMC4wLjAwMDAiLCJQIjoiV2luMzIiLCJBTiI6Ik1haWwiLCJXVCI6Mn0%3D%7C3000%7C%7C%7C&sdata=HeUBWX3v%2FHiBl3hJ9p9Mw2AvvdLlW5NfieYhBQRflDI%3D&reserved=0)
Natural Earth (public domain): [http://www.naturalearthdata.com/](https://eur03.safelinks.protection.outlook.com/?url=http%3A%2F%2Fwww.naturalearthdata.com%2F&data=05%7C01%7Ce.sebire.21%40abdn.ac.uk%7Caf6ba56de2fd45b8d16b08dbeb4fd3d7%7C8c2b19ad5f9c49d490773ec3cfc52b3f%7C0%7C0%7C638362500315978973%7CUnknown%7CTWFpbGZsb3d8eyJWIjoiMC4wLjAwMDAiLCJQIjoiV2luMzIiLCJBTiI6Ik1haWwiLCJXVCI6Mn0%3D%7C3000%7C%7C%7C&sdata=osmV1RZF7wcofZYwqI%2BNHQ99NRkpeer6D6%2BhjBTbZfU%3D&reserved=0)

Thank you for your comment. Mapchart.net does have a CC BY 4.0 licence, and in the terms and conditions of the website, the publisher states:

‘*This work is licensed under a* [*Creative Commons Attribution-ShareAlike 4.0 International License*](http://creativecommons.org/licenses/by-sa/4.0/)*.You are allowed to use, edit and modify any map (image) created with the Website or Mobile App for personal, non-commercial, or commercial use/publication freely, by adding any kind of reference to the Website or Mobile App in your work.’*

We had included a ‘created with Mapchart.net’ in the figure legend, but we have now amended this in line with your advice to make the licence clearer:

‘Map created using Mapchart.net under a CC BY 4.0 license, with permission from Minas Giannekas, original copyright [2023].’ [Figure 2 legend, line 226]

2. **Reviewer #1**

Comment:

In the field of fetal DNA floating in maternal peripheral blood, the acronym NIPT has to be indicated at least once as cffDNA-NIPT in papers where various non-invasive testing methods are analyzed.

Thank you for taking the time to review our manuscript and for providing your comments. We agree with the need to clarify the NIPT acronym. The use of cell-free foetal DNA is contested, as the DNA is of placental rather than foetal origin. Whilst placental and foetal DNA are usually the same, this is not always true, for example in cases of confined placental mosaicism. This is an important limitation when using NIPT to inter foetal genotype. We have therefore clarified the NIPT acronym in the introduction as follows:

‘*NIPT detects and characterises cell-free DNA (cf-DNA-NIPT), that is shed from the placenta into the maternal circulation,…’* [Line 65].

The described background of this paper is the basis of all global government programs for the reduction of invasive genetic diagnosis for Down syndrome. In such global scenario the data evaluation criteria must include also the ethnic, legal and ethical differences that strongly influence the application of the tests and the decision-making aspect of the pregnant woman.

Thank you for your recommendation. We agree that the wider legal, religious, and cultural factors will strongly influence the offer of antenatal screening within specific countries, and women’s responses to screening. We had added the following comment to reflect this in the introduction:

‘*Screening for DS is part of routine antenatal care in many countries (3). However, the offer of screening, its uptake by women, and women’s diagnostic and reproductive choices following a high chance screening result, vary between countries and population subgroups, reflecting wider legal, religious, and cultural factors.’ (New text underlined) [Line 54]*

We also already include the following statement in the discussion:

‘*Overall, vast heterogeneity in the healthcare systems and cultural differences between populations and regions will strongly influence the use and uptake of NIPT. What is clear is that each country has implemented NIPT in a different way, depending on several factors that might include the pre-existing infrastructure for a screening programme; how the healthcare system runs; expected cost of healthcare; the cultural and societal expectations on pregnancy screening and acceptability of disability in these populations’. [Line 482]*

We did attempt to collect data on key sociodemographic factors that may influence the uptake of screening such as maternal age and ethnicity, however this data was not provided consistently in our included papers and therefore could not be accounted for in meta-analyses.

Taking on board your comment, we have added the following table summarising current legislation on termination of pregnancy in each of the 27 countries/autonomous regions included in part A to our supplementary material:

**S2 Table: Termination of pregnancy laws in part A included countries/autonomous regions.**

| **Country / state / province** | **Termination of pregnancy (TOP) law** | | **Reference** |
| --- | --- | --- | --- |
|  | **Legal/restricted** | **Gestational limits for TOP** |  |
| Hong Kong | Legal: 1. If continuing pregnancy would risk the pregnant woman’s mental or physical health 2. If the baby will be born with a severe disability | 24 weeks | Family Health Service, Department of Health, The Government of Hong Kong Special Administrative Region (1) |
| Japan | Legal: social and economic grounds (spousal authorisation required) | No limit stated | * |
| Victoria, Australia | Legal: on request | 24 weeks  (beyond 24 weeks must be approved by two medical practitioners) | * |
| Switzerland | Legal: on request | 12 weeks | * |
| Taiwan | Legal: social or economic grounds (required: spousal authorisation, permitted for foetal diagnosis) | Not reported | * |
| Ontario, Canada | Legal: on request | 24 weeks | National Abortion Federation, Canada (2) |
| Sweden | Legal: on request | 18 weeks | * |
| Belgium | Legal: on request | 14 weeks | * |
| Denmark | Legal: on request | 12 weeks | * |
| South Spain – Andalucía | Legal: on request | 14 weeks  (22 weeks in cases of risk to maternal life or fetal disability) | * |
| The Netherlands | Legal: on request | No limit stated | * |
| Yukon, Canada | Legal: on request | 12 weeks | National Abortion Federation, Canada |
| Wales, UK | Legal: broad social and economic grounds | 24 weeks  (No gestational limit in cases of risk to maternal life or fetal disability) | * |
| Poissy Saint-Germain, France | Legal: on request | 16 weeks | * |
| Poland | Restricted: only cases of rape, incest, or to preserve maternal life. | N/A | * |
| Romania | Legal: on request | 14 weeks | * |
| Iceland | Legal: on request | 22 weeks | * |
| Lithuania | Legal: on request | 12 weeks | * |
| Italy | Legal: on request | 90 days | * |
| Finland | Legal: on requestion | 12 weeks | * |
| Slovenia | Legal: on request | 12 weeks | * |
| USA | Restricted: varies by state (as of June 2022) abortion is now regulated at the state level and has been heavily restricted in states across the U.S e.g., very early (6 week) gestational limits on TOP or prohibited except when ‘necessary to preserve life’. | Where legal, varies by state | * |
| Korea | Legal: on request | No limit stated | * |
| Moscow, Russia | Legal: on request | No limit stated | * |
| Scotland, UK | Legal: broad social and economic grounds | 24 weeks  (No gestational limit in cases of risk to maternal life or fetal disability) | * |
| England, UK | Legal: broad social and economic grounds | 24 weeks  (No gestational limit in cases of risk to maternal life or fetal disability) | * |
| California, USA | Legal: on request | No limit stated | * |
| Norway | Legal: on request | 12 weeks | * |

Table to describe the legal guidelines around termination of pregnancy in each of the included countries/autonomous regions included in part A. The laws have been categorised into ‘legal’, ‘restricted’ or ‘illegal’, with further information about the access to legal/restricted terminations of pregnancy in that population. * Information sourced from the Centre for Reproductive Rights World Abortion Laws map – accessed from The World's Abortion Laws - Center for Reproductive Rights (3).

We have also added the following to the discussion:

‘When analysing the uptake of screening, and the prevalence of live births and terminations of pregnancy within our included studies, it is important to consider the variation in legal and wider cultural factors between the countries/regions represented that may influence findings. For example, in Table S2, we present the current termination of pregnancy laws for the areas represented in part A of our review. Substantial variation between areas under which termination is permitted, and related gestational limits, is evident. Although we are not able to formally investigate any specific association between termination of pregnancy laws and the quantitative outcomes measured in part B, it is likely that variation in legal and wider cultural factors explains, at least in part, the variation in outcomes and heterogeneity present in our meta-analyses.’ [ Line 491]

The quantitative amount of the case-sample used for the analysis must be presented in the text with the numerical values of the cases alongside the percentages, referring only the details to the tables.

Thank you for your comment. We have added the numerical values into the percentages described in the results, with reference to the details in the tables:

*Data was extracted from five studies reporting the proportion of women opting for IPD pre-NIPT and post-NIPT implementation.*

*‘In the pre-NIPT period, values ranged from between 48% (117 out of 243) and 93% (235 out of 253) of women who had a high chance biochemical screening result for DS, that opted for IPD. A meta-analysis of proportions was conducted to produce a random effects pooled estimate for the proportion of women with a high chance pregnancy opting for IPD (Fig.4A) indicating a pooled proportion of 75% (95% CI 53%, 88%, I^2^ = 97%) of women that underwent IPD in the period before NIPT was introduced.’ (New text underlined) [line 312]*

*‘After NIPT was available in these populations, 30% (72 out of 243) to 62% (474 out of 763) opted for IPD after a high chance biochemical screening result, the pooled proportion was 43% (95%CI 31%, 56%; I2 = 95%), suggesting that the proportion of women opting for IPD following a high chance biochemical screening is reduced after NIPT is implemented as a second line screening test in the same population (Fig.4B).’ (New text underlined) [line 316]*

The decision of the pregnant women in terms of therapeutic abortion or DS livebirts is not relevant for the aim of the study, so the number of abortions and affected births cannot be the subject of meta-analysis but only of analytical and documented description by patient's age, gestation period, legal regulations of the country.

Thank you for this comment. The aim of our paper is to examine the global introduction of NIPT into national screening programmes for prenatal Down’s syndrome screening and the potential impact this may have on decisions made during pregnancy. As such, we have outlined four main outcomes: NIPT uptake, invasive prenatal diagnosis uptake, and live births and terminations of pregnancy for babies with Down’s syndrome. These represent relevant outcomes of high interest to policy makers, clinicians, and families.

The outcomes of live births and termination of pregnancy for babies with Down’s syndrome are important as an indicator of pregnancy outcomes following NIPT implementation. As noted in our response to the previous comment, we agree that wider legal and cultural factors will influence women’s reproductive choices following a prenatal diagnosis of Down’s syndrome. However, it is also possible that the type of screening offered (e.g., introduction of NIPT) independently influences women’s choices. This is the primary focus of our study and we therefore think it is important to report on these outcomes both at a country- and pooled-level (from meta-analyses).

The data of 27 selected papers, a small number, is more eligible for a traditional review than a meta-analysis, to be able to appreciate and not lose specific characteristics of each study. The great amount of limitations of the study described by the authors in their conclusions demonstrates that the meta-analysis does not allow to produce added value to the study exceeding some intuitive practical conclusions.

Thank you for this comment. In total, we identified 41 papers for inclusion in this systematic review. For part A, 21 papers were included after meeting the inclusion criteria, and are described in detail in table 3 – providing information on 27 countries that have implemented national NIPT for Down’s syndrome. 31 met the criteria for part B (10 of which are also in part A) and are included in meta-analyses.

We have followed the guidance from Cochrane on systematic reviews, which states:

*‘Meta-analysis is appropriate when the following criteria can be met by the studies of interest: • All the outcomes are comparable and can be pooled meaningfully. • All the interventions and comparators are the same, or at least similar enough to be combined meaningfully. • The correct data are available for the included studies; for example, means and standard deviations are necessary to analyse a continuous outcome (Note: even if the results are not reported in this format by the study authors, mean and standard deviation can often be calculated from the data provided). Two studies is a sufficient number to perform a meta-analysis, provided that those two studies can be meaningfully pooled and provided their results are sufficiently ‘similar’. [Ryan R; Cochrane Consumers and Communication Review Group. ‘Cochrane Consumers and Communication Group: meta-analysis. http://cccrg.cochrane.org, December 2016 (accessed 1/12/23)]’*

Our data fit these parameters – we have sufficient studies for each analysis (at least n=5), have grouped our meta-analyses by data relating to comparable populations and outcomes, and explored and identified sources of heterogeneity through appropriate and comprehensive sensitivity analyses and meta-regressions. We acknowledge that there are wide confidence intervals and high heterogeneity between studies. However, these results reflect the data that is available currently and still provide important information for the reader and stakeholders on the decisions made by women following NIPT implementation. They emphasise the importance of producing comparable data between populations for continual and robust monitoring to be done. Not reporting these results might also contribute to publication bias. We were unable to extract consistent information regarding specific sociodemographic characteristics of each study population, despite undertaking an exhaustive search of published and grey literature databases. Similar limitations in terms of difficulty extracting comparable contextual information from source studies is commonly encountered in systematic reviews.

We acknowledge that the outcomes we report are sensitive, and therefore we have found it important to emphasise the limitations of each meta-analysis to the reader. By informing the reader of the study limitations we are being as transparent as possible. Although we have gone into detail about the limitations, this review also has many strengths and we have clarified these as follows:

*‘This systematic review followed focussed review questions, with inclusion and exclusion criteria selected a priori and published on the PROSPERO register. It utilised an exhaustive search strategy on multiple databases, using sources that delivered both published materials and grey literature. A standardised risk of bias assessment tool was used, with appropriate subgroup, meta-regression and sensitivity analyses to comprehensively explore sources of heterogeneity and bias in the included studies. The screening of manuscripts was undertaken by two independent reviewers, and discussion taken to a third reviewer for settling disagreements. The main outcomes of interest; uptake of NIPT, uptake of invasive prenatal diagnosis, termination of pregnancy, and live births of babies with Down’s syndrome reflect relevant outcomes of high interest to policy makers, clinicians and families and we are able to provide important information on the overall trends in decision making within populations where NIPT has been introduced.’ (new text underlined) [line 447]*

Thank you again for all the important points you have raised.

3. **Reviewer #2**

Comment:

In this study the authors do a systematic review to understand the extent of NIPT introduction for Down’s syndrome into national screening programmes worldwide, its uptake among eligible populations and the impact it may have on specific pregnancy outcomes.
As reported by the authors themselves, this review has many limitations because many studies used for the meta-analyses do not report all the data necessary to analyse the outcomes of interest.
Furthermore, the paper has some points to be reviewed.

1. Some points regarding NIPT need to be corrected:
- DNA sequencing methods are not the only technologies used in non-invasive prenatal testing (lane 12-13), there are other technologies such as microarrays, nanofiltration plates with fluorescence scanning, Droplet
Digital PCR (ddPCR)

Thank you for bringing this point to our attention, we have edited the relevant section in the introduction and added text as follows:

*‘NIPT detects and characterises cell-free foetal DNA (cf-DNA-NIPT), that is shed from the placenta into the maternal circulation. by using high throughput sequencing methods such as chromosome-selective sequencing, massively parallel shotgun sequencing and single-nucleotide polymorphism based approaches (Lo et al.1997, D'Aversa et al., 2022, Gil et al., 2017). These sequencing methods quantify the proportion of each chromosome in the maternal plasma and identify differences from the expected distribution. An excess or deficit in the expected chromosome profile would indicate a foetal trisomy or monosomy respectively (Hui and Bianchi, 2020).’ [Line 68]*

- re-evaluate literature reference 6 in lane 58;
We have updated our original reference 6 (Lo et al., 1997) in line with the additional information shown in the paragraph above including the new references D’Aversa et al., 2022, Gil et al., 2017 and Hui and Bianchi, 2020. [Line 71]

- false-positive cases may be indicative of placental mosaicism, missing twins and "presence of maternal chromosomal abnormalities", the latter condition may be suggestive of maternal cancer, but not only (lane 71).

Thank you for bringing this to our attention, we agree that our statement should be made more accurate when referring to the indications of a false positive NIPT result. We have amended this in the manuscript to:

‘*indicative of placental mosaicism, vanishing twin, or maternal chromosomal abnormalities which could suggest maternal cancer (15). [line 91]*

2. In Figure 2 and in the text, the authors do not explain all the reasons why 125 studies out of 167 were excluded from the review (30 are excluded due to 'unsuitable study population', 42 due to 'experimental study design' and the remaining 53?).

We thank you for highlighting this error – our original PRISMA diagram had not uploaded properly, and therefore the other reasons for exclusion were not visible in the flow diagram. We have amended this and the reasons for exclusions for all 125 papers are now given. The figure is shown below:

3. In Table 3, the authors refer to the Gadsboll et al. 2020 paper, but do not detail the situation in the individual countries reported as they should. For example, in Italy there are official guidelines supporting the use of NIPT in high-risk women already before 2019, but only some regions, such as Tuscany or Apulia, currently reimburse the test.

Thank you for your feedback. Our systematic review was a thorough search of published literature, including grey literature databases, following the inclusion and exclusion criteria we had decided a priori. As such, we were only able to extract data from those studies that have met the inclusion criteria, and unable to capture unpublished information that has not been picked up by our systematic search strategy. We have revisited Table 3, amending it to make sure the information from Gadsboll et al is captured as accurately as possible for each country that has a national or regional offer of NIPT – this study data was provided through responses from clinical experts and as such, has not been included in further analyses.

The changes to this row in table 3 are shown below, where the row has been expanded to give specific information on each country where this could be extracted from Gadsboll et al., 2020:

| Country/state/province | Year of NIPT implementation | When is NIPT offered in the pathway? | Funding | Risk threshold for accessing NIPT | Reference |
| --- | --- | --- | --- | --- | --- |
| Poland, Romania, Iceland, Lithuania, Italy,  Finland, Slovenia,  USA | Not reported | Poland and Romania - second line screen | Patient funded | 1:100 – 1:1000  (invasive testing offered to those with a chance above 1:100) | Gadsboll et al 2020* |
|  |  | USA - Second line screen  NIPT not offered under Medicaid for 9 States. | Medicaid coverage described for NIPT in many USA states | Not reported |  |
|  |  | Iceland – Second line screen. Higher chance women can access NIPT over invasive testing if requested. | Publicly funded | Not reported |  |
|  |  | Italy – Second line screen | Patient funded  Some regions publicly funded: Tuscany, Bolzano | Not reported |  |

**Data repository**

Data to be publicly available in repository.

Dataset is stored in the OSF project directory, anonymous link (view only) here:

<https://osf.io/85qg4/?view_only=179cbc7b4acc4a1cbbcf19d6b8dca3b5>

Thank you again for all the important points you have raised.
